# Constructing future behavior in the hippocampal formation through composition and replay

**DOI:** 10.1038/s41593-025-01908-3

**Published:** 2025-03-10

**Authors:** Jacob J. W. Bakermans, Joseph Warren, James C. R. Whittington, Timothy E. J. Behrens

**Affiliations:** 1 Wellcome Centre for Integrative Neuroimaging, University of Oxford, Oxford, UK; 2 Department of Basic Neuroscience, University of Geneva, Geneva, Switzerland; 3 Sainsbury Wellcome Centre for Neural Circuits and Behaviour, University College London, London, UK; 4 Department of Applied Physics, Stanford University, Stanford, CA USA; 5 Wellcome Centre for Human Neuroimaging, University College London, London, UK

**Keywords:** Learning algorithms, Decision, Hippocampus

## Abstract

The hippocampus is critical for memory, imagination and constructive reasoning. Recent models have suggested that its neuronal responses can be well explained by state spaces that model the transitions between experiences. Here we use simulations and hippocampal recordings to reconcile these views. We show that if state spaces are constructed compositionally from existing building blocks, or primitives, hippocampal responses can be interpreted as compositional memories, binding these primitives together. Critically, this enables agents to behave optimally in new environments with no new learning, inferring behavior directly from the composition. We predict a role for hippocampal replay in building and consolidating these compositional memories. We test these predictions in two datasets by showing that replay events from newly discovered landmarks induce and strengthen new remote firing fields. When the landmark is moved, replay builds a new firing field at the same vector to the new location. Together, these findings provide a framework for reasoning about compositional memories and demonstrate that such memories are formed in hippocampal replay.

## Abstract (web summary)

A model of compositional state spaces in the hippocampus shows latent learning and rapid generalization. Bakermans et al. show that this model predicts the emergence of place responses in replay.

## Main

A recent spate of the hippocampal models suggests that hippocampus represents a state space and its transitions, which form a cognitive map^1–4^. These models come in the following two flavors: those that infer the state space from sequences^3,4^ and those that use the state space for reinforcement learning (RL)^1,2^. Together they explain many key hippocampal findings, from associative learning in neuroimaging studies^5,6^ to precise cellular responses during spatial sequences, such as place^7^ and grid cells^8^. Additionally, they account for latent state representations in RL tasks that require nonspatial behavior, such as splitter cells^9,10^ in spatial alternation tasks or lap cells^11^ in tasks that require counting^3,4^. Overall, these models’ successes have been greatly suggestive that the hippocampus builds state spaces from sequences and may use these state spaces for RL.

However, these new ideas about state-space inference seem at odds with key principles of hippocampal function that are supported by a wealth of empirical evidence. Most strikingly, hippocampus’ primary role is one of memory^12,13^. This memory is part of a constructive process that also supports imagination and scene construction and understanding^14–19^. This evidence suggests that the hippocampal representation is compositional, binding cortical information together to build a representation of the current experience. A door to the northwest. A wall to the south. A friend sitting at the table. Indeed, empirically, hippocampal neurons respond to conjunctions of external features^20^, as if binding together elements of a composition, and RL state-space tasks predominantly rely on hippocampus when new state spaces are initially constructed^21^. The big computational benefit of compositionality is being able to understand and respond to situations in one shot. This flexibility is missing from the traditional state-space models. For those models, learning how states relate to each other requires observing state transitions. This often requires much experience and can be brittle to policy or local transition changes^22^. These observations raise a substantial puzzle—how do the state-space models relate to the well-known memory and construction machinery of hippocampus?

Here we unify the two through a model of state-space composition. To be compositional, the model of the world must be decomposable into representational sub-blocks (*z* = (*z*^1^, *z*^2^, *z*^3^, …)), with independent sub-block dynamics (${z}_{t}^{i}=g({z}_{t-1}^{i})$). This means that new configurations of the sub-blocks (corresponding to a new world model) have predictable dynamics with no extra learning. Instead of learning state-space transitions from experience, these transitions can now be inferred. In this scenario, the world model for any particular situation specifies the current building blocks combination—a role we propose for hippocampal place cells. (We refer to the cortical building blocks as compositional as they can occur in any configuration, but we propose the composition of these building blocks occurs in hippocampus.) For example, composing a map of space with an object-centric map (or wall- or door-centric) means the hippocampal state space knows where the object (or wall or door) currently is in space. This feature of hippocampal compositions offers several advantages beyond hippocampus being just state spaces or memories alone and provides new insights into hippocampal phenomena. We show that (1) conjunctive hippocampal cells can be reinterpreted as compositionally binding together multiple maps/variables; (2) there is a dramatic performance gain (versus standard RL) when hippocampal state spaces are compositions of already learned building blocks, because policies learned on one hippocampal composition generalize to new compositions; (3) replay can compose states spaces offline into memories that improve future behavior either by updating policies or consolidating existing memories and (4) this constructive function of replay affords precise predictions of how, when and where replay changes hippocampal representations. Indeed, when testing these predictions in single-unit recordings in the rodent hippocampus, we find that (1) replay induces and strengthens hippocampal place fields—new place fields emerge after replay events and rate map changes align on replayed locations; (2) these rate map changes reflect structural elements as well as rewards and (3) the resulting responses generalize compositionally.

## Results

### Model of hippocampal compositions

**Fig. 1:**
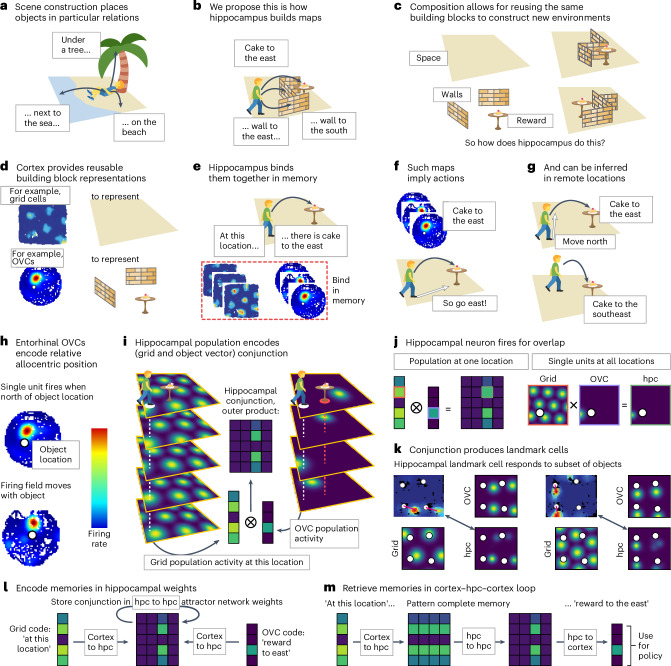
Model of hippocampal state-space composition. **a**, To construct a scene, like this (imagined) experience of being on the beach, hippocampus binds objects into relational configurations. **b**, We propose that hippocampus similarly composes state spaces from structural elements like walls and rewards (black arrows denote allocentric vector relations). **c**, The advantage of composition is that new situations, like new spatial environments, can be constructed from the same building blocks. **d**, Cortex can provide such reusable representations, for example, in the form of grid cells^8^ and OVCs^25^ in spatial environments. **e**, Hippocampus can construct new situations from cortical representations by binding them together in relational memory. **f**, Because these compositional state spaces are built from reusable elements, understanding from one environment generalizes to others. In particular, these maps immediately imply actions (white arrow). **g**, To construct the state space, building block representations can be propagated to remote locations—online, but also offline in replay. **h**, OVCs^25^ encode the global relational knowledge that supports action inference. If this example cell fires and the object is a reward, you should go south. **i**, Hippocampus then combines this information, for example, from the four OVCs for reward on the right, with a spatial code, for example, from the five grid cells on the left, into a new conjunctive memory encoded in 20 hippocampal cells, for example, through an outer product. **j**, The outer product describes the population activity; on the single-neuron level, this produces place responses in hippocampus—these are place cells that carry reward information. A single unit in hippocampus (green outline) is activated when its grid cell input (red outline) and OVC input (purple outline) overlap. **k**, The same mechanism can produce hippocampal landmark-vector cells (empirical, top left^32^; simulated, bottom right) that respond to some objects in the environment but not to others. These two examples show how the conjunction of the grid cell (bottom left), with firing fields on a hexagonal grid, and an OVC (top right), with firing fields and a fixed vector relation to each object (white circle), produce a hippocampal response (bottom right) that is similar to an empirically measured response (top left). **l**, These conjunctive representations are encoded in hippocampal memory through recurrent hippocampus–hippocampus weights. In this way, hippocampus stores the link between two cortical inputs (for example, a grid code (left) and object-vector code (right)). **m**, Such a memory can then be retrieved from partial representations. For example, a cortical grid representation can index the hippocampal representation, which pattern completes the encoded memory through the recurrent hippocampal connectivity so that the corresponding object-vector code is retrieved in cortex. This retrieved object vector serves as input to the policy—if there is a reward to the east, you should probably go east. Icons in panels **a**–**g**, **i** were adapted from Twemoji, under a CC BY 4.0 license. The grid cell ratemap in panels **d**,**e** was adapted from ref. ^8^, Springer Nature Limited. The OVC ratemap in panels **d**–**f**, **h** was adapted from ref. ^25^, Springer Nature Limited. OVCs, object-vector cells; hpc, hippocampus.

We propose that hippocampus uses reusable building blocks that are composed together to understand new situations—just like scene construction (Fig. 1a). For clarity, we explain this model through spatial representations, but it applies whenever the world dynamics can be split into compositional parts. In this section, we give a high-level overview of the model and its main features, which we will further elaborate on in the following sections. The Methods and Supplementary Information provide implementational details.

In space, to construct a scene of a simple room, hippocampus may bind together a representation of where you are in space, *x*, with where you are relative to walls (*w*), salient objects (*o*) and rewards (*r*; Fig. 1b). Critically, the subcomponents (*x*, *w*, *o* and *r*) are reusable because any room is a different configuration of space, walls, objects and rewards. Thus, the hippocampal state space can be built immediately, for any new environment, out of different conjunctions of these building blocks (Fig. 1c). This idea of composition through hippocampal conjunctions is not new^23^; indeed, composing space with observations has been shown to support relational inference and structural generalization^4^. However, there is a crucial difference in the conjunction of space and structural building blocks proposed here—these structural building blocks afford generalizing policies. The resulting hippocampal representations do not just map the world—they specify how to act in it.

A key question, however, is what the building blocks should look like. Fortunately, for space, walls, objects and rewards (*x*, *w*, *o* and *r*), biology already tells us. In the entorhinal cortex and hippocampus, along with place and grid cells that code for space, there are vector cells that point toward walls, objects and rewards—border-vector cells, object-vector cells and reward-vector cells^24–27^. Each cell provides a distance and direction to the border/object/reward so that the population of vector cells provides a map that can be path integrated (updated with respect to actions taken), just like grid cells for space, but rather with each map being centered around the border/object/reward (Fig. 1d). These cells generalize and so are reusable—an object-vector cell in one environment is also an object-vector cell in another environment, just like grid cells.

The reusability of these building blocks means any understanding of one configuration can be generalized to new configurations. This is particularly powerful for RL, as now an agent does not have to learn a new policy from scratch for every new environment (like RL on conventional hippocampal state spaces, for example, the successor representation^1,3^). Instead, the compositional state space already implies actions (Fig. 1f). A reward-vector cell says head toward the reward, a border-vector cell says do not crash into the border and so on. In RL terminology, credit assigned to these compositional building blocks in one situation is also useful in new situations; that is, the hippocampal state space comes ‘pre-credit-assigned’. This means online RL is dramatically reduced and often not necessary at all.

But how can this composition be achieved in the first place? Given the compositional state representation, the policy can be generalized—but upon entering a new environment, it must first be constructed. That requires binding representations of (*x*, *w*, *o* and *r*) at every location in the appropriate configuration, that is, binding the vector representation *r* saying ‘3 steps north to the reward’ to the spatial representation *x* when you are actually three steps south of the reward and storing it as a hippocampal memory (*x*, *r*) (Fig. 1e). Therefore, in principle, the compositional state space can be constructed step by step in online behavior. However, performing compositions like that is slow because it requires visiting all states of the new environment. It is also error-prone as it relies on path integration^28,29^. Fortunately, biology provides a potential resolution in the form of offline replay^30^, which can bind building blocks together in remote locations (Fig. 1g). This is both more data efficient and reduces potential errors. In this context, replay is effectively performing credit assignment because it constructs the state space for future successful behavior; the next time we are at that remote location, we already know about the reward.

### Place cells for global knowledge in local representations

The constructive nature of hippocampal function suggests a particular interpretation of hippocampal place cells—a conjunctive representation^20,31^, where hippocampal cells bind existing representations into a new relational configuration. To be a useful state space for inducing behavior, the local (at a given location) representation must contain global (about other locations) relational knowledge (Fig. 1f). Therefore, hippocampal representations must be conjunctions between cortical cells that encode this global relational knowledge, such as object- and border-vector cells in the medial entorhinal cortex (Fig. 1h). With such a representation, unlike with existing place cell models, many transitions are inherited rather than learned as they are implied by the particular combination of cortical inputs.

So what do these conjunctive representations look like? We assume that the hippocampal population encodes the outer product of the representations it composes together (Fig. 1i). The resulting single-unit responses exhibit spatial tuning just like place cells (Fig. 1j)—but these are place cells that carry reward information. Such responses have been found empirically in the form of hippocampal landmark-vector cells^32^, with place fields at vector relations for some objects in the environment but not others (Fig. 1k). We propose that these conjunctive representations are stored in hippocampal memory through recurrent hippocampal connectivity (Fig. 1l). Once the memory is encoded, it allows for using one cortical representation, for example, a grid code of where you are, to retrieve another, for example, an object-vector code of the relative location of reward (Fig. 1m). The latter can serve as input to a policy for navigating toward that reward.

Notably, these conjunctions need not be the only hippocampal representations and can be further tuned as the environment becomes familiar or behavior is overlearned (for example, to build a successor representation). Their power comes from their potential to generate behavior immediately in a new environment.

### Compositional codes facilitate zero-shot behavior

**Fig. 2:**
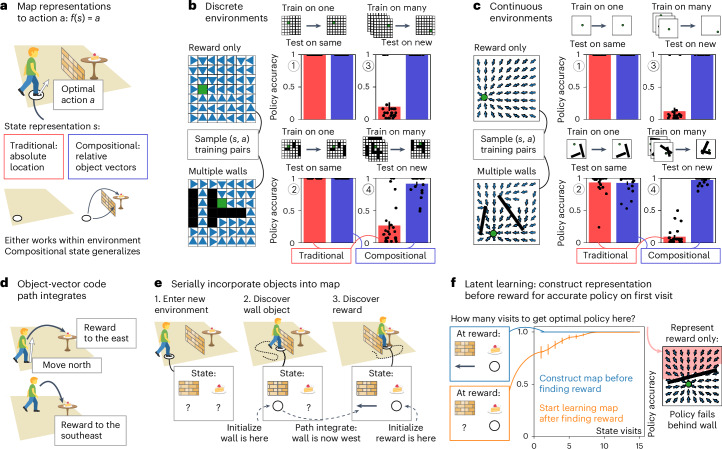
Compositional state representations generalize and can be built through latent learning. **a**, We learn a mapping, *f*, from state representation, *s*, to optimal action, *a* (white arrow)—*f*(*s*) = *a*. For a given location, *s* represents the absolute location in the environment (traditional; red, *s* = (*x)*) or the relative vector codes (black arrows) for all objects (walls and rewards) in the environment (compositional; blue, *s* = (*w*, *r*)). **b**, Policy accuracy at test locations for traditional (red) and compositional (blue) representations in simple reward-only (top) and complex multiwall (bottom) discrete environments. In discrete graph environments, we sample (state representation and optimal action (blue triangles)) training examples, both in simple environments with reward only and in complex environments with multiple walls. When trained on a single environment and tested on the same environment, both the traditional and compositional state representations provide accurate policies (panels 1 and 2). Only mappings from compositional state representations yield accurate policies when tested in a new environment (panels 3 and 4). *n* = 25, error bars = s.e.m. **c**, Policy accuracy at test locations for traditional (red) and compositional (blue) representations in simple reward-only (top) and complex multiwall (bottom) continuous environments. In continuous environments, where locations are continuous coordinates and actions are continuous directions, we find the same results. Either representation works within environments (panels 1 and 2), but only the compositional state representation generalizes (panels 3 and 4). *n* = 25, error bars = s.e.m. **d**, How to obtain this compositional state representation in new environments? The vector code (black arrow) that makes up these representations path-integrates. Because it follows relational rules independent of the environment, the representation can be updated with respect to the agent’s action (white arrow). For example, if a reward is to the east and the agent goes north, the reward is now to the southeast. **e**, Path integration allows for serially incorporating objects (or walls or rewards) into the compositional map. The object-vector code is initialized on object discovery and then carried along as the agent explores the environment. **f**, Policy accuracy at states behind the wall with respect to reward as a function of the number of visits to those states, when learning structure from the start (blue) or after finding reward (orange). Our agent thus learns about the structure of the environment without being rewarded. Once they find a reward, this latent learning allows access to optimal actions on the first visit to locations behind the wall (blue). Without latent learning, the agent needs to rediscover the wall to obtain the optimal policy behind the wall (orange). Error bars: s.e.m. Icons in panels **a**, **d**–**f** were adapted from Twemoji, under a CC BY 4.0 license.

To explore this potential for zero-shot generalization, we compare two agents (Fig. 2a). In both cases, we train a feedforward network, *f*, through supervised learning on ground-truth optimal policies to predict optimal actions, *a*, given a state representation, *s*. For the first agent (traditional), states are unrelated to environmental features—they only represent space, *x*. For the second agent (compositional), states are compositions of vector cells to environment features—they combine walls, objects and rewards (*w*, *o* and *r*). We do not include space (*x*) as part of this composition as it is not required for action selection (but we will need the spatial component later to build compositional maps in memory). In each case, we sample multiple environments with walls, objects and rewards, placed at random. The optimal policy is defined as the local actions that minimize the number of steps to reward from each location. We then sample (state representation and optimal action) pairs (*s* and *a*) as training examples to train a feedforward network that maps state representations to optimal actions through supervised learning. We evaluate the network’s performance by measuring whether following the learned policy successfully navigates to the goal from a set of test locations (Policies that generalize).

While the traditional agent can learn arbitrarily complex policies in a single environment with the reward in a fixed location (Fig. 2b_1 and b_2)), it immediately fails when either the reward or environmental features change (Fig. 2b_3 and b_4). This is unsurprising as it is not retrained, and the state representation does not carry useful information across environments. By contrast, the compositional agent immediately generalizes behavior. Given the state representation contains reward vectors, this is trivially true for changes in reward location in an otherwise empty environment (Fig. 2b_3). However, it also holds for policies that require complicated trajectories avoiding multiple walls (Fig. 2b_4). These findings hold across both discrete (Fig. 2b) or continuous (Fig. 2c) state and action representations. Together, these results emphasize that if hippocampal cells form a state space for learning policies, they should build on existing relational structures (for example, from cortical building blocks), instead of being learned from scratch.

Compositional representations therefore allow for dramatic behavioral generalization. In this view, place cells bind together cortical representations into a memory, such that when the animal next visits this location, they will be able to reactivate cortical representations that link to optimal actions. Paradoxically, because the agent does not need to have taken the action to build the memory, this is a memory of future behavior. This memory of the future permits zero-shot inferences.

### Latent learning and laying down memories of the future

Compositional reasoning, therefore, changes the computation required to produce good behavior in a new environment. Instead of learning a new behavioral policy, we must lay down new memories. But critically, we must lay down these memories everywhere in the environment. This is important because encountering a new wall will change the optimal policy far beyond adjacent states. When the agent arrives at a remote location, its state representation must reflect whether there is a wall between the agent and the reward.

Like credit assignment in RL, we therefore must update state information at remote locations. But these updates carry structural information rather than reward expectations. Instead of value, we must transfer the newly discovered compositional features to each state in the environment. Critically, in compositional worlds, the independent parts of the representation can be updated independently. In space, object-centric representations can be path-integrated independently of allocentric representations (Fig. 2d). If an agent is one step east of a reward and takes a step east, it is now two steps east of the reward. Hence, one approach is simply to keep track of the path-integrated representation of each element encountered while traversing the environment. Better still, building these representations into memories alleviates the requirement to keep track of many variables at the same time. As the representation is compositional, environmental features can be added one at a time.

This process of serially integrating information into a compositional state representation naturally accounts for results in latent learning, where animals with experience of the environment without rewards rapidly develop optimal policies upon discovering rewards for the first time^33^. Similarly, when our compositional agent explores the environment, it builds an increasingly complete state representation, *s*, incorporating objects as they are encountered (Fig. 2e). Then the agent finds a reward. In a simulation where the agent has already learned about the wall-vector representation, it only needs a single visit (to add the goal vector to *s*) to any other state for access to the optimal policy (Fig. 2f, blue). Without this latent learning, the state representation does not include the wall yet, so the agent needs to rediscover it to accumulate the full state representation, *s*, and optimally avoid the wall (Fig. 2f, orange).

### Replay builds memories efficiently

**Fig. 3:**
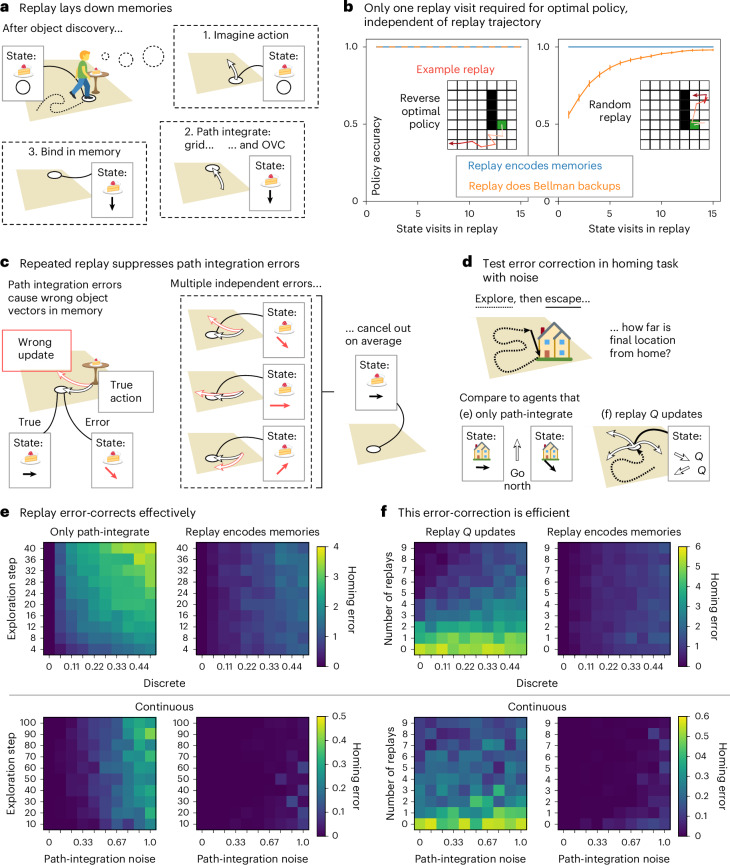
Replay builds compositional maps in memory. **a**. After discovering an object (or wall or reward), replay builds the compositional map in memory by path integrating the vector code and the grid code and binding them together to form new hippocampal memories at remote locations. **b**, Policy accuracy as a function of replay state visits for replay trajectories that follow the reverse-optimal policy (left) and random replay trajectories (right) for replays perform credit assignment (orange) and replays that encode memories (blue). The latter kind of replay (blue) provides optimal policies in a single replay visit. On the other hand, replay that performs Bellman backups for credit assignment (orange) requires a single visit for optimal actions only if the replayed trajectories follow the opposite optimal policy (left) but needs many more replay visits for random replay trajectories (right). Error bars = s.e.m. **c**, Path integration is noisy. This can be mitigated by forming memories in multiple different replays because path integration errors are independent and average out. **d**, We test the error-correcting capacity of replay that encodes memories in a homing task with path integration noise. The agent starts from home, explores the environment and then needs to escape back home as quickly as possible. We compare the agent that encodes memories in replay to an agent that only path-integrates a homing vector (**e**) and an agent that replays *Q* updates to learn which actions are expected to lead toward home (**f**). **e**, Homing error when varying the length of exploration (*y* axis) and path-integration noise (*x* axis), for a path-integrating agent (left) and an agent that additionally replays to encode memories (right), in discrete (top) and continuous (bottom) environments. For the agent that path integrates only (left), the homing error (distance from home after escape) increases for longer exploration and higher path integration noise. For the agent that encodes home-vector representation memories in replay, the error is greatly reduced. **f**, Homing error when varying the number of replays (*y* axis) and path-integration noise (*x* axis), for an agent that replays *Q* value updates (left) and an agent that encodes memories in replay (right), in discrete (top) and continuous (bottom) environments. The agent that encodes home-vector memories in replay needs fewer replays to achieve lower homing errors than the agent that replays *Q* updates. Icons in panels **a**,**c**,**d** were adapted from Twemoji, under a CC BY 4.0 license.

In the previous section, we argued that path integration allows for serially incorporating objects into a compositional map during online exploration. However, if the agent must construct representations through path integration by physically traversing the environment and can process only one or a few representations at a time, then it will need to traverse the environment many times to build a latent representation. To avoid this problem, rather than physically path-integrating vector representations, the agent can imagine path integration in replay (Fig. 3a). In this interpretation, replay can achieve credit assignment by exactly the same mechanism as it uses to build memories—through the conjunction of cortical building blocks.

This leads to clear, untested predictions. Replay events should happen in the vector cells when an animal discovers a new environmental feature (such as an object or a reward). But critically, the replay events must bind object-centered representation to their correct locations in allocentric space. This means that allocentric representations must also path integrate in replay, simultaneously with the object-vector cells. A natural candidate for this allocentric representation is the grid cell representation, which is known to path integrate^34,35^. We therefore predict that replay events will involve simultaneous replay of grid cells and object-vector cells, where both cell populations replay to the same locations but in two different coordinate systems—global and object-centric, respectively (Fig. 3a). As the conjunction of the two will be encoded in hippocampal memory, hippocampal replay trajectories should be coherent with these (as observed in ref. ^36^; however ref. ^37^ reported independent replay). This also implies the resulting hippocampal conjunctions (in this case, a hippocampal object-vector-grid conjunction; landmark cells) at given locations can be active before ever physically visiting that location. Empirically, a landmark cell can be detected from its activity during online behavior. We predict that some landmark cells will appear in replay after discovering the corresponding object before they appear in physical navigation.

Notably, with noiseless path integration, the optimal new policy is constructed with a single visit to each state, whatever the trajectory of the replay (Constructive replay). This is unlike alternative interpretations of replay—if replay performs Bellman backups^38^ (as in the Dyna algorithm^39^), it can only update the value of any state by comparing to its neighbor, so it is exquisitely sensitive to the trajectory of the replay. To achieve convergence with a single step would require prescience (Fig. 3b, left), as replay would need to play out (in reverse) the new optimal policy that results from the updated values. Unlike Bellman backups, replaying for compositional memory requires only a single visit to each state, even if the trajectory is random (Fig. 3b, right). This is because path integration through a world model (spatial or otherwise!) is trajectory-independent, and so the elements that get composed at each state are identical regardless of the replay’s trajectory.

However, the reliance on path integration introduces a vulnerability—path integration is noisy, and errors accumulate over time. Replay will therefore build noisy, but unbiased, memories. This noise can be reduced with repeated replays (Fig. 3c). One attractive feature of this proposal is again that it aligns the replay’s role across credit assignment and memory. Here multiple replays consolidate the existing memory^40,41^.

We simulate a noisy homing task where the agent explores an arena starting from a home location until it encounters a threat and needs to return home as quickly as possible (Fig. 3d; Constructive replay). Without replay, the homing error for the path integration agent increases both for higher noise and for longer exploration, as path integration errors accumulate (Fig. 3e, left, top for discrete domains, bottom for continuous). With replay, homing errors of both causes are dramatically reduced (Fig. 3e, right). Notably, Bellman backups are also susceptible to path integration errors, as backups can be attributed to incorrect states. This can be ameliorated by sampling replay repeatedly (Fig. 3f, left). Nevertheless, when replay is instead used to build compositional memories, the smaller homing error is achieved with fewer replays (Fig. 3f, right).

The proposed constructive function of replay affords a new normative theory of prioritized replay (Supplementary Information—Optimal replay creates memories where they matter most) to predict replay patterns. It is also readily extended to hierarchical or nonspatial construction, as hippocampus does empirically, with consequences for hierarchical RL (Supplementary Information—Composing nonspatial and hierarchical building blocks). Both are omitted here due to space constraints, but we direct the interested reader to the Supplementary Information or ref. ^42^.

### Empirical evidence of replay for map-making

Instead, we will now turn to validate our model by testing our (preprint–preregistered^42^) neural predictions. In particular, we will test whether ‘some landmark cells will appear in replay (…) before they appear in physical navigation’ in two datasets (Testing replay predictions). We find that upon replay, changes in the rate map of hippocampal neurons (1) closely align to the neuron’s replayed location, (2) reflect structural changes beyond just reward and (3) generalize in a compositional way. Together, these results support a role for replay in building maps (1) from structural elements (2) through composition (3). We demonstrate how replay changes maps in hippocampal recordings during an alternating home-away well task^43^. When we decode replay trajectories while the animal is at the home well, where it needs to return later, we discover that rate map changes occur exactly where a cell fires in replay. Moreover, for certain cells, these rate map changes generalize across home well locations, affording compositional reconfiguration when the environment changes. Finally, such environment changes may include structure beyond reward—in a four-room maze task where doors lock halfway through the experiment^44^, we find new place fields that emerge after replay at a recently locked door.

**Fig. 4:**
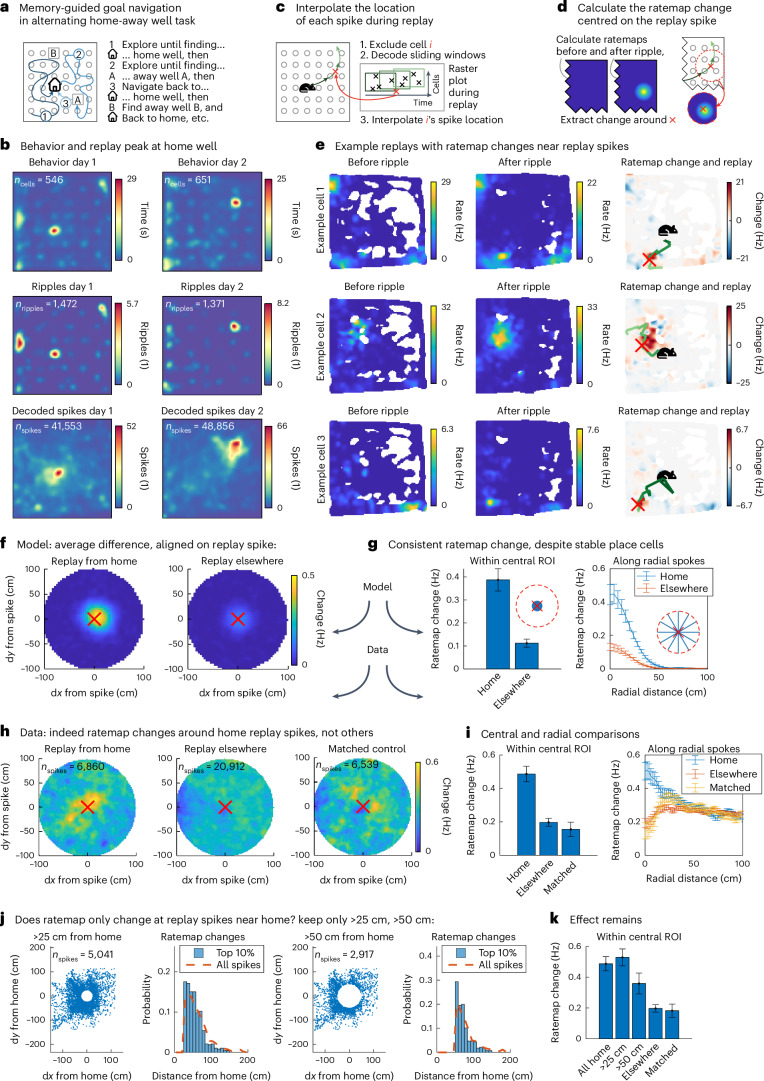
Replay changes hippocampal rate maps. **a**, In the alternating home-away well task, the animal needs to alternate between exploring for the currently rewarded random away well and navigating to the remembered stable home well. **b**, Heatmaps of occupancy (first row), ripple locations (second row) and interpolated spike locations (third row) across animals on day 1 (left) and day 2 (right) of the experiment. The occupancy averaged across animal peaks at the home well location (first row), and many sharp wave ripples occur there (second row). Within the ripples, interpolated spike locations are distributed across the arena (third row). **c**, To calculate these interpolated spike locations, we estimate the location of a spike of cell *i* during replay by decoding the replay trajectory while excluding cell *i*, then interpolating the spike time along the replay trajectory. **d**, We then measure the rate map before and after that replay event for a and calculate the rate map change aligned on its replay spike location. **e**, Rate maps of three example cells before a ripple (left column), after a ripple (middle column) and the difference between the two (right column) with the decoded replay trajectory (green) and the interpolated replay spike (red cross) overlaid. These example cells show a large positive rate map change (right column; red, positive and blue, negative change) near the interpolated replay spike (red cross) along the decoded trajectory based on all other cells (green line, from dark to light). **f**, Rate map change aligned on interpolated replay spike location averaged across neurons and replays in simulated hippocampal neurons. We find a consistent change at the replay spike that is stronger for replays from home that create new landmark fields (left) than replays elsewhere that do not (right). **g**, Rate map change of simulated neurons, averaged across replays and neurons, near (left) and extending from (right) the interpolated replay spike location. Averaging the aligned simulated change maps within an 8 cm region of interest at the replay spike location (left), or along radial spokes extending out 100 cm in uniformly sampled directions from the replay spike location (right), further illustrates the same effect. We find a consistent rate map change despite the inclusion of place cells with stable rate maps. *n* = 753 (home) and *n* = 1,143 (elsewhere); error bars = s.e.m. **h**, Rate map change aligned on interpolated replay spike location averaged across neurons and replays in recorded hippocampal neurons. In the same analysis of empirical data rather than simulations, we find the same result. On average, the rate map changes align on the replay spike for home replays (left), but not for replays elsewhere (middle), also after matching them for time within the experiment (right). **i**, Rate map change of recorded neurons, averaged across replays and neurons, near (left) and extending from (right) the interpolated replay spike location. Averaging the aligned change maps within a narrow central region of interest (left) or along radial spokes (right) again highlights this effect. *n* = 6,860 (home), *n* = 20,912 (elsewhere) and *n* = 6,539 (matched); error bars = s.e.m. **j**, Locations of included replay spikes with respect to the home well (left) and histogram of home well distances for the replay spikes that produce the greatest 10% of rate map changes (right) when excluding spikes within 25 cm (first) and within 50 cm (second) of the home well. It is not just the replay spikes near home that contribute to these rate map changes, as evidenced by excluding replay spikes within 25 cm (left) or 50 cm (right) from home. Some of the largest rate map changes occur in remote locations. **k**, Rate map change of recorded neurons, averaged across replays and neurons, near the interpolated replay spike location (as in *i*). After keeping only remote (>25 cm and >50 cm) replay spikes, the effect of rate map changes at the replay spike remains. *n* = 6,860 (all home), *n* *=* 5,041 (>25 cm), *n* = 2,917 (>50 cm), *n* = 20,912 (elsewhere) and *n* = 6,539 (matched); error bars = s.e.m. Mouse icon was created by Freepik on Flaticon and house icon was created by DazzleUI on svgrepo under a CC BY 4.0 license. Source data

We probe how replay changes maps in hippocampal recordings collected in a study discussed in ref. ^43^ in an alternating home-away well task (Fig. 4a). This task involves an arena where reward appears in hidden reward wells, following a very specific pattern—first at a fixed ‘home’ well, followed by a random ‘away’ well, then at the same fixed home well again, then a different random away well, and so on. The animal thus needs to alternate between random exploration to discover the currently rewarding away well, followed by memory-guided navigation to return to the home well. According to our model, this task can be solved through a hippocampal home-well map that provides this return policy. Our theory proposes that this map is constructed from reusable vector representations in replay. We therefore need to test how replay changes hippocampal representations. This paradigm is ideal for that—the animal returns to the home well many times, providing ample opportunity for map-making replays; meanwhile, the search for the random away well yields good behavioral coverage of the arena, to probe what that map looks like.

We identify replay events by detecting sharp wave ripples (SWRs) in the local field potential. We first observe that, although previous analyses of this data have focused on ripples during navigation^43^, in fact, many ripples occur when the animal is at the home well (Fig. 4b). Are these ripples modifying the hippocampal map in a compositional way? We ask whether spatial neural responses change after a replay, and where that change happens. If replay is indeed responsible for creating new conjunctions, we expect a neuron’s rate map to change at the exact location where that neuron fired in the replay event because the replay event path integrates relational knowledge around the map. To find out, we need to know where a cell fired in a replay event in a way that is not contaminated by its own rate map. To achieve that, we decode the animal’s position from their neural activity in sliding windows through the replay, but we do this decoding in a slightly convoluted way—we repeat the decoding for each neuron that spikes during the replay event while excluding that neuron, then interpolate the location of its spike (Fig. 4c). So how does the rate map change—what is the difference in the neuron’s rate map before and after the replay event—around that location (Fig. 4d)?

We find a strong effect of aligned positive rate map changes at the replay spike location, specific to replays from the home well. When we apply the procedure described above to such replay events, we discover hippocampal cells that form a new place field right around the interpolated spike location (Fig. 4e, green line (decoded replay trajectory) and red cross (interpolated spike location)). To understand how such emerging place fields look across a population of recorded neurons, we simulate replay-induced map changes in a synthetic neural population of hippocampal place cells (Supplementary Fig. 11c) and landmark cells (Supplementary Fig. 11a,b; Home-well mapping simulations). Our simulation shows that for replays that build maps, on average, we expect a small but consistent increase in firing rate right at the location of the replay spike (Fig. 4f, left). The increase is consistent because of our simulated landmark cells, which first fire in replay and then obtain a new receptive field. However, the increase is small because it is averaged across many neurons (including our simulated place cells, with stable rate maps) and replays (whereas only the first replay creates a new field from scratch). Nevertheless, the resulting aligned change will be greater for replays from home that lay down new vector representations than for replays elsewhere that do not (Fig. 4f (right) and Supplementary Fig. 12). We show the same effect in a different way by averaging the changes within an 8 cm radius around the replay spike (Fig. 4g, left) and by averaging across spokes in every direction from the replay spike (Fig. 4g, right).

Indeed, when we average the rate map change around the replay spike across cells and replay events in the dataset of ref. ^43^, we find a strong rate map increase right at the replay spike—for replays from home, but not for other replay events (Fig. 4h; matched control = selection of replay events elsewhere with a time distribution matched to the replays from home). We use these replays elsewhere as controls because we expect them to contribute less to map changes, as the new landmark cells are already present before the away well replays. Averaging the resulting change maps within a narrow region around the replay spike, or along radial spokes outwards from the replay spike, further illustrates these differences (Fig. 4i). This effect holds even when only considering replay spikes more than 50 cm from the home well (Fig. 4j,k and Supplementary Fig. 7).

However, these responses could have been reward-related because there is a reward at the wells. Our theory posits that the hippocampal map also incorporates other elements, like walls. We test if replay maps such structural elements—here, blockades—in recordings provided in ref. ^45^ in a four-room configurable maze task^44^. In this task, the animal needs to navigate to one of four rooms connected by doors, forage there and then navigate to a different room, and so on. Halfway through the experiment, certain doors lock without changing their appearance—the only way to find out if a door is locked is by trying it. A locked door has major implications for the maze topology, so we expect the animal to incorporate this change in the hippocampal map through replay on discovery.

We collect rate maps before and after the doors close and look for replay when the animal discovers this structural change (Supplementary Fig. 8a). Because we cannot detect SWRs or decode replays in this dataset, we define replay at doors through ‘nonlocal door spikes’ (Supplementary Fig. 8b). A nonlocal door spike occurs when a neuron fires outside any of its place fields while the animal sits at a closed door and serves as a proxy for a replay that propagates the obstacle vector. We identify cells in which such replay precedes the emergence of new place fields (Supplementary Fig. 8c) and find that across the population, cells that replay are more likely to get new place fields (Supplementary Figs. 8d (linear regression, one-tailed *t*-test) and 9). Because of the indirect replay measure here, this second set of results should be interpreted with some care; they are suggestive, but to be conclusive will require a dataset where enough cells are recorded simultaneously to decode replay trajectories.

**Fig. 5:**
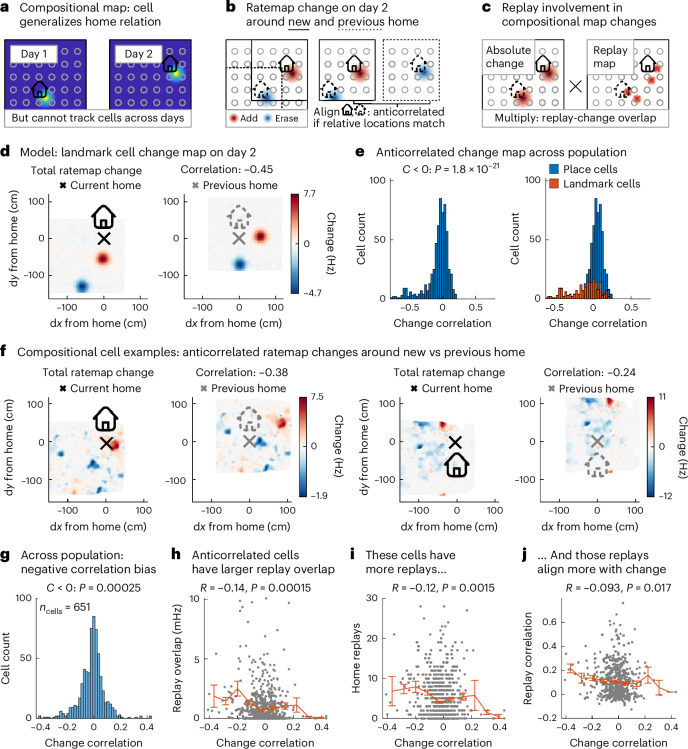
Rate map changes generalize. **a**, In a compositional map, home responses generalize so that new home-well configurations can be mapped by recombining existing elements. **b**, To detect generalized responses without access to cells across multiple days, we plot the rate map change of a cell on the second day and ask if the change with respect to the new home is anticorrelated to the change with respect to the previous home. **c**, If replay is involved in such changes, the cell’s replay spike locations should overlap with the rate map changes. **d**, Example rate map change on day 2 of a simulated landmark cell whose object-vector cell fires to the south of the home well. When we align the same change map first on the new home well location (left, black) and then on the old home well location (right, gray), the positive and negative changes overlap so the maps anticorrelate. **e**, Histogram of correlation between current-home and previous-home aligned rate map change of simulated hippocampal neurons. Across the population of simulated hippocampal neurons, vector responses produce a correlation histogram with a negative bias (left, one-tailed *t*-test against zero). Splitting the contributions of simulated place and landmark cells (right) highlights that the landmark cells are the source of this negative bias. **f**, Two examples of recorded neurons with anticorrelated changes (red, positive and blue, negative) with respect to the new (left, black) and previous (right, gray) home. **g**, Histogram of correlation between current-home and previous-home aligned rate map change of recorded hippocampal neurons. Across the population, this rate map change correlation is negatively biased, suggesting a subpopulation of neurons with generalized home responses (one-tailed *t*-test against zero). **h**, Overlap of replayed locations and rate map changes plotted against change correlation (same *x* axis as **g**) for each neuron. The rate map changes of anticorrelated-change neurons occur at similar locations as their replay spikes (linear regression, one-tailed *t*-test; gray, cells and orange, average of *y* values within ten bins along the *x* axis). **i**, Number of home replays against change correlation (same *x* axis as **g**) for each neuron. These anticorrelated-change neurons have more replays. **j**, Overlap of replayed locations and rate map changes against change correlation (same *x* axis as **g**) for each neuron. The rate map change locations of anticorrelated-change neurons correlate more with their replay locations. **g**–**j**, *n* = 651 and error bars = s.e.m. House icon was created by DazzleUI on svgrepo under a CC BY 4.0 license. Source data

The previous results support a role for replay in map-making. For this map to be compositional, cells need to generalize their home response across environments (Fig. 5a). For example, in the dataset from ref. ^43^, a home-vector cell would fire southeast of the home well, regardless of its location (which changes across days). Hippocampus can then recombine such representations to construct new maps of any home-well configuration by binding these vector representations to specific locations. This conjunction produces hippocampal landmark cells (Fig. 1k) with firing fields at the same spatial relation for the home well on day 1 and day 2. Can we find evidence of these generalized home responses?

Not only do we find neurons that show such responses, but we also discover that for these neurons, replay correlates with rate map changes. While we cannot track cells across days, we can probe the cumulative change of each neuron’s rate map on day 2 (Fig. 5b). If a neuron generalizes, it will gain a new place field at a fixed spatial relation to the new home—but it should also lose the place field at that same relation to the previous home. That produces a measurable signature—the rate map change aligned on the current home should anticorrelate with the rate map change aligned on the previous home. Our simulation of neural responses to the home well on different days recovers this signature of generalized home well responses. The example simulated landmark cell in Fig. 5d fires when the animal is to the south of the home well. Therefore, the increase in firing rate in red on day 2 (Fig. 5d, left) aligns with the decrease in firing rate in blue on day 2 with respect to where the home well used to be on day 1 (Fig. 5d, right), resulting in a negative correlation of the shifted change map. Our simulation shows that this produces a negative bias in the correlation coefficients (Fig. 5e, left), due to the consistent remapping of landmark cells (Fig. 5e, right).

Indeed, we find neurons with anticorrelated-change maps (Fig. 5f), and on the population level, there is a negative bias in the aligned change correlation (one-tailed *t*-test; Fig. 5g). To investigate replay involvement, we also ask whether the location of the replay spikes for a neuron overlaps with these rate map changes (Fig. 5c). It turns out that these cells on the left of the distribution are also the ones with the highest replay overlap (linear regression, one-tailed *t*-test; Fig. 5h)—they have both more replay spikes (Fig. 5i), and the locations of replays and rate map changes correlate (Fig. 5j)—which suggests that replay may be responsible for these changes. Taken together, these results indicate that within the population of hippocampal cells with spatial responses, there is a subpopulation of landmark cells (the ones that receive input from entorhinal vector cells, according to our model) that exhibit vector responses that may be constructed in replay.

## Discussion

Recent results have suggested a path toward building a formal understanding of neural responses in flexible behaviors. By assuming that the hippocampal formation builds a state space (cognitive map) from sequential observations, these models have not only revealed computational insights into the hippocampal involvement in RL^1,2^ but also accounted for a variety of single-neuron responses^3,4^. However, while this has the potential to bring formal explanations to an array of new scenarios, it is not clear how these models relate to classic hippocampal functions such as episodic memory^13,14^, scene construction^15^ or imagination. Indeed, hippocampal patients are impaired not only in navigation but also in scene recognition^46^ and imagination of future and fictitious scenes^16,17,47^. In this work, we propose a framework where these disparate functions can be expressed in the same formal language. We formalize hippocampal state spaces as compositions of reusable building blocks. We have shown that this affords flexibly generalizing behavior to new situations on the first encounter by simply rearranging previously learned components.

We propose that hippocampal cells provide conjunctions of prelearned building blocks, specifying their arrangement in the current experience. This means that hippocampus no longer needs to learn transitions itself, as these are inherited from the building block dynamics. Furthermore, we show that forming memories of these compositions during exploration provides the ideal state space for future behavior when observing the reward (latent learning). Notably, if the building blocks have a forward model, then memories can be formed offline in replay. This enables an agent to efficiently build a compositional state space for future behavior. This constructive interpretation of replay naturally extends to changes in task structure as well as reward and integrates these ideas with the compositional nature of hippocampal/entorhinal representations.

Our empirical data support this compositional mechanism in two complementary datasets. In ref. ^44^, there are many examples of the first discovery of new landmarks (here closed doors), allowing us to show that cells with new firing fields were likely to have fired in replay when the door was discovered. In ref. ^43^, there were fewer first-discovery events but many visits to the landmark (here a home well). We showed that each replay event changed the place map at exactly the inferred location where the cell fired during replay. Furthermore, we showed that these changes are compositional. When the landmark moves, replay builds a new place field at the same relative location, but now to the new landmark. Together these findings suggest that when a new structural element is discovered in the world, replay lays down a compositional memory that embeds the vector to this landmark. We note that an earlier version of this paper on bioRxiv^42^ included this as a preregistered test of the theory. We would like to thank the reviewers for encouraging us to perform this test.

Computational models, in particular REMERGE^23^ and Tolman–Eichenbaum machine (TEM)^4^, have previously shown that conjunctive coding in hippocampus supports generalization. However, the type of conjunction and the scope of generalization as a result are fundamentally different here. REMERGE learns links between sensory features within an environment through sensory–sensory hippocampal conjunctions. That allows for sensory generalization, like transitive inference, within but never across environments, because there is no abstract state-space structure embedded in the hippocampal representation. TEM does learn abstract structure to make sensory–structural hippocampal conjunctions, which allow for sensory generalization across environments. But in our model, the state-space structure itself is compositional. By forming structural–structural conjunctions, we compose state spaces from structural building blocks. As a result, we can generalize behavior—we make inferences about actions, not sensory input. Rather than zero-shot predicting what the agent will see, as in TEM, we zero-shot predict what the agent will do. Because that requires knowledge of global relational structure (for example, a wall to the east and a reward to the south), this global information must be propagated to the local hippocampal representation. That is exactly what we propose replay is for.

Notably, we do not propose that all composition in the brain requires hippocampus. Composing sentences from words or motor skills from primitives is not impaired after hippocampal damage. In fact, even generalizing policies from structural building blocks here does not require hippocampus—the building blocks are represented in cortex. But hippocampus becomes essential when these cortical representations need to be stored in relational memories. This is exactly the same machinery as episodic memory or scene construction, or other compositional tasks that hippocampus is involved in—hippocampus constructs new representations from whatever the relevant inputs are. We therefore argue that not all composition takes place in hippocampus, but the hippocampal machinery for relational memory and replay turns out to be remarkably powerful for making compositional maps that generalize policies.

This minimal role of replay and memory for hippocampus is appropriate when worlds can be composed perfectly from existing primitive building blocks. By contrast, in models where hippocampus learns the environment’s transition structure^1,3^, any transitions can be modeled (after learning). One intriguing possibility is that both systems can be invoked. Compositional inference gives rapid, flexible approximate behavior, which is nuanced by modeling of transitions. In such a system, another possible role for replay is to build new cortical primitives when experiences have been poorly modeled by the existing repertoire.

While we have elucidated a role for replay in the online setting, this framework also suggests a neural interpretation of two previously proposed roles for replay in sleep^48^—building cortical primitives (as above) and learning the policy on the compositional state space. Here replay could help learn compositional policies by generating training examples for policy mapping *f*. By sampling random vector representations for walls and rewards, the agent dreams up arbitrary new environment configurations and trains the policy mapping from simulated trajectories. This is a natural extension to the idea of learning an inverse model during sleep (Helmholtz machine^49^), but it is particularly powerful for compositional forward models as ‘sleep training’ can include samples that have never been experienced^48^.

Extreme generalization by composition is a fundamental property of human and animal cognition. While this has been self-evident in cognitive science for many decades, it is typically ignored in computational neuroscience. In this work, we have taken this notion seriously and tried to align it with a series of properties of hippocampal function—some long-known (memory, construction and conjunctive coding) and some more recently discovered (state spaces for controlling behavior). As the community attempts to find formal descriptions for computations underlying increasingly rich and complex behaviors, we believe that compositional reasoning will play an increasingly important role in future models, not only of cognition but also of neural responses.

## Methods

A Python implementation of all models and simulations is available at https://github.com/jbakermans/state-space-composition. Here we first describe the modeled worlds and agents, in general, and then provide details of each figure’s simulations.

### Discrete and continuous worlds

We implement agents in continuous and discrete environments. Although similar in principle, these two types of agents and environments are slightly different in their practical implementation. The discrete agent behaves on a graph, whereas the continuous agent behaves in a two-dimensional (2D) Euclidean space. Both discrete and continuous settings are deterministic Markov decision processes (MDP).

The discrete environments are defined by a set of locations and actions as in a deterministic MDP. All results here assume rectangular square grid worlds, with actions ‘north’, ‘east’, ‘south’ and ‘west’. We generate environments by adding walls and rewards on top of these regular grids. We represent rewards with a population of object vector cells, where each cell fires at a specific vector in relation to the reward, that is, a cell that fires 1 step to the East and so on. We represent each wall with two populations of object vector cells—each vector population centered on one of the wall ends. In more detail, we calculate the object vector population activity at location *x* relative to object *o* by concatenating the vectors of one-hot encoded distance from *x* to *o* along each action, with −1 distance for actions in the opposite direction. For example, for an object, one step east and three steps south from *x*, the representation is concatenate(onehot(−1), onehot(1), onehot(3), onehot(−1)). Thus, at any location, there are four vector cells active in the whole population—one for each action. In square grid worlds, this representation has redundancy because east is the opposite of west and north the opposite of south, but this setup allows for accommodating any type of nongrid graph too.

In continuous environments, locations become continuous (*x*, *y*) coordinates and actions are steps in a continuous direction. We place walls and rewards within a square 1 m × 1 m arena, at random locations and orientations. Again, we represent rewards by a single population of object vector cells and walls by two populations, one centered on each wall end. A single object vector cell is defined by a 2D Gaussian firing field, tuned to a specific distance and direction from its reference object; the firing fields of the full object vector cell population are distributed on a square grid centered on the object. We thus calculate the object vector population activity at location *x* relative to object *o* by evaluating the population of Gaussians centered on *o* at *x*.

### Agent: tracking representations

Our agent does not have access to these vector representations when it enters a new environment (except in Fig. 2a–c, where we provide the full state representation). It needs to discover objects first. During exploration, the agent observes its location and initializes a vector representation on object discovery, that is, sets concatenate(onehot(0), onehot(0), onehot(0), onehot(0)) at that location *x*. From then on, it updates the vector representation on each transition. This update combines the following two components: (1) it path integrates its previous vector representation with respect to its action and (2) it retrieves any existing vector representations previously stored in memory based on the current observed location (the memory links vector representations to location representations). It then stores the updated representation at the new location in memory.

To implement this process, we use two practical abstractions from a full neural system. First, we use a direct location signal (an ID of the location), instead of a neural grid code from which the location is traditionally thought to be decoded. Second, we instantiate memory as a key-value dictionary, rather than a hippocampal attractor network that stores conjunctions (these are in fact directly relatable to each other^50^). In this dictionary, the space representation serves as the key and the object/wall/reward vector as the value, so that stored vector representations can be retrieved at a given location. These abstractions do not change any model principles but keep the implementation simple.

The discrete agent initializes an object’s vector representation when it is at a location adjacent to it. Then on each step, it path integrates the representation—but due to path integration noise, the represented vector relation might diverge from the true vector relation. We model this noise as a probability distribution *p*_PI_ over the updated representation so that it reflects either the correct transition with probability (1 − *e*_PI_) or one of the neighbors with probability *e*_PI_. In addition to path integration, as stated above, the agent relies on memory to update its vector representation. After observing its new location, it retrieves vector representations inferred there previously. That produces another probability distribution *p*_*M*_ overrepresented vector relations, with the probability of a vector representation proportional to the number of retrieved memories of that representation. The agent then samples the final updated representation from the weighted sum of the path-integrated and memory-retrieved distributions—*s* ~ *w* × *p*_PI_ + (1 − *w*) × *p*_*M*_. Finally, it stores the sampled representation in memory at the new location.

The continuous agent tracks representations in a similar fashion. It discovers an object when it comes in range (5 cm), initializes the corresponding vector representation and from then on updates it after every step. We model path integration errors in continuous space by adding Gaussian noise to the direction and step size of the update to get a path-integrated vector representation *s*_PI_. Again, the agent combines this path-integrated representation with a representation that is retrieved from memory. Continuous memories store vector representations at continuous locations; the agent retrieves all memories created within a cutoff distance (5 cm) from its new location and then weights the vector representations stored in these memories with a softmax over the cosine similarities between the memory location and the new location to obtain *s*_*M*_. The updated representation is the weighted sum of path integration and memory retrieval—*s* = *w* × *s*_PI_ + (1 − *w*) × *s*_*M*_. The agent then encodes this inferred representation in memory at the new location.

### Conjunctive memories

We propose that hippocampal representations are conjunctions of building block representations and that these conjunctions can be stored as memories in hippocampal weights (Fig. 1). While it is possible to implement a conjunctive representation in different ways, here we use an outer product representation; that is, the conjunction *c* between representations *a* and *b* will have cells corresponding to the product of all pairs of *a* and *b* cells. So if *a* has 3 cells and *b* has 4 cells, then *c* will have 12 cells. To demonstrate what conjunctive representations (for example, between spatial representations and sensory representations) would look like in hippocampal recordings, we simulate populations of grid cells, object vector cells and sensory neurons. Each of these neurons is defined by one or multiple spatial Gaussian firing fields, arranged on a triangular grid (grid cells), at a fixed distance and direction from an object (object vector cells), or at a random environment location (sensory neurons). See example conjunctive representations in Fig. 1.

To show how hippocampal landmark cells could result from conjunctions of object vector cells and grid cells (Fig. 1k), we generate object vector cell rate maps and perform the outer product with a grid cell representation. This leads to landmark cell responses because the grid cell’s peaks do not always align with every object (or object vector cell), and so each conjunctive cell will not necessarily be active around every object.

The TEM^4^ proposed a specific biological implementation of this conjunctive memory. The authors model place cells as conjunctions of grid cells in the medial entorhinal cortex and sensory codes in the lateral entorhinal cortex. They use a Hopfield network with fast Hebbian plasticity to implement the hippocampal memory. The result is an auto-associative attractor network. The recurrent dynamics of such a network allow for pattern completion of partial representations so that one part of the conjunction can retrieve the other part. This means that observing the current state of the environment supports inference of the corresponding grid code. Or, the other way around, access to the grid code of a location allows for predicting the corresponding observation. We hypothesize a similar neural implementation of conjunctive memories here, except that the conjunctions combine structural building blocks. However, our actual simulations will mostly abstract away this biologically plausible setup. Instead, we implement conjunctive memories through a simple ‘key-value’ dictionary, where one part of the conjunction acts as the ‘key’ to index the other part of the conjunction as the ‘value’ for encoding and retrieval (Agent: tracking representations; Supplementary Information—Mapping the environment).

### Policies that generalize

If an agent has access to the full compositional vector representation, it should be able to behave optimally even if it has never seen the particular composition before (Fig. 2a–c). We demonstrate this by learning a policy mapping *f*(*s*) = a that maps an input state representation to an output optimal action. The compositional state representation that serves as input to this policy mapping concatenates the vector representations as described in the Discrete and continuous worlds for all objects in the environment. When there are multiple objects in the environment, like a reward and two walls, the representations of the objects are concatenated in a consistent order, for example, first the reward and then the two walls. This is important because a reward has different consequences for behavior from a wall; between the walls, the order does not matter. Effectively this means the agent encodes vector relations for different objects in different populations of object-vector cells and knows the identity of each object. An alternative but equally viable implementation could use a separate object-vector population for each object type, with multiple activations for multiple objects of the same type. For the environment with only a reward (Fig. 2b_1, b_3, c_1 and c_3), the input representation consists of just a reward-vector code, and for the environment with multiple walls, the input representation concatenates a reward-vector code and three (in discrete environments; Fig. 2b_2 and b_4) or two (in continuous environments; Fig. 2c_2 and c_4) wall-vector codes. Here we always provide complete representations (which contain all objects in the environment) as input to the policy mapping, but if objects are missing (for example, when they do not exist in the current environment or when they have not been identified yet), their representations can be set to zeros to mimic a population of object-vector cells without activity. The output action is a vector of probabilities across the four discrete actions in discrete environments; in continuous environments, the output action is a 2D vector that contains the sine and cosine of the optimal direction. For the compositional vector representation, *s* is the concatenation of object vector population activities described above (for example, rewards and walls). As a control, we also learn a mapping for a representation of absolute location (traditional), which is a representation that does not know about walls or rewards.

We implement the function *f* as a feedforward neural network, with three hidden layers (dimensions 1,000, 750 and 500 in discrete environments; 3,000, 2,000 and 1,000 in continuous environments) with rectified linear activations. For the wall representation (which is two populations of vector cells per wall), we include an additional single network layer (common to all walls) that takes in the two populations and embeds them (same embedding dimension as input dimension) before feeding them into the first hidden layer of *f*—this is not necessary for learning but does speed it up. We then sample (state representation and optimal action) pairs as training examples from environments with just a reward and environments with a reward and multiple walls and train the network weights in a supervised manner through backpropagation using PyTorch’s autograd and the ADAM optimizer with default hyperparameters. To evaluate a learned mapping, we sample locations and then simulate a rollout that follows the learned policy and calculate the fraction of locations from where following that policy leads to reward (within 5 cm).

To test whether policies learning from a single environment generalize (Fig. 2b_1, b_2, c_1 and c_2), we sample training examples from one environment (discrete, 1,000 samples; continuous, 2,500 samples) and test on the same environment for 25 environments independently. To test whether policies learned from many environments generalize (Fig. 2b_3, b_4, c_3 and c_4), we train the network on 25 environments in parallel (batched input), sampling training examples in each environment (discrete, 200 samples; continuous, 500 samples) before sampling a new set of 25 environments (100 times). We then test the learned mapping for a new set of 25 environments not included in the training.

### Latent learning

Upon entering a new environment, we allow the agent to obtain and update compositional representations of objects/walls even in the absence of reward—this is latent learning (Fig. 2e,f). To show the utility of latent learning (Fig. 2e), we simulate agents with and without latent learning in a discrete environment with a wall and reward. We consider the situation where both agents have already explored the environment and found the wall, and now they have just discovered the reward. The agent that does latent learning then has a full vector representation of wall and reward, while the agent without latent learning only knows about the reward vector (as it did not obtain or update the wall representations when it saw the wall earlier). We simulate both agents as they continue their exploration. The latent learning agent can use path integration to continue updating wall/reward-vector representations and can use these to calculate the optimal policy, wherever it goes even if it has never been there before. The nonlatent learning agent, on the other hand, needs to rediscover the wall to incorporate it in its state representation before it can behave optimally from all locations. To calculate the difference in their optimality, for each location behind the wall (where the full vector representation is required for appropriate behavior), we calculate whether the agent would be able to successfully navigate to the reward based on its current representation on every encounter. We average policy success across these locations on the 1st, 2nd, etc., until the 15th encounter and repeat the simulation 25 times in different environments.

### Constructive replay

Replay offers a way of carrying vector representations to remote locations without having to physically navigate there (Fig. 3). During replay, the agent imagines actions, path integrates location (grid cell) and vector (object vector cell) representations and binds them together by encoding a new memory of the resulting combination. We model these replayed transitions like the ones in physical navigation, with the following two important differences: (1) the agent cannot observe the transitioned location like in behavior, so there can be path integration errors in the memory location (the ‘key’ in the dictionary) as well as in the representation (the ‘value’), and (2) it only relies on path integration to update representations during replay, without the memory retrieval.

First, we compare an agent that encodes memories in replay like this to an agent that instead carries out credit assignment by temporal difference learning through *Q* updates (Fig. 3b). We apply ‘backward’ *Q* updates (temporal discounting factor *γ* = 0.7 and learning rate *α* = 0.8) in the opposite direction of replay (that is, after a replay transition from *a* to *b*, we calculate a backup from *b* to *a*) to make credit assignment more efficient. We sample replay trajectories that start from the reward location and either extend out along a random policy (Fig. 3b, right) or a reverse-optimal policy (Fig. 5b, left). We then calculate for each step in the replay trajectories whether the currently learned policy, either according to the encoded vector representation or the *Q* values, provides an optimal path to reward from that step’s location. We aggregate policy success by the first, second, etc., until the fifteenth replay visit to each location and average across locations and repeat the simulation 25 times in different environments.

Then, we investigate the consequences of path integration noise (Fig. 3e,f). We simulate a homing task, where the agent starts from home and initializes its home-vector representation, then explores the arena, until it needs to escape to home as quickly as possible. During exploration, the agent builds home-vector memories by replaying from the home location five times every four steps and retrieves these memories when it is updating its current home-vector representation (that is, using memories to reduce path integration noise). During the escape, it selects actions that lead home according to its current home-vector representation. In two different experiments, we compare the agent to two controls. The first control agent-only path integrates its homing vector, without encoding or retrieving any memories (and without any replay). In this experiment (Fig. 3e), we vary path integration noise and the total number of steps during exploration. The second control agent carries out *Q* updates to learn actions (discretized direction in the continuous environment) expected to lead home in on-policy replays and uses the learned *Q* values to find the way home during escape. Because the current location can be observed from the environment, the read-out of *Q* values during escape does not suffer from path integration errors, but path integration noise does affect the replayed *Q* updates—during replay, the location to update *Q* values needs to be path integrated, so credit can get assigned to the wrong place. In this experiment (Fig. 3f), we vary the path integration noise and the number of replays that the agent engages in every four steps.

### Testing replay predictions

We test our replay predictions—in particular, replay builds maps, or more prosaically, ‘some landmark cells will appear in replay (…) before they appear in physical navigation’—in the following two datasets: the alternating home-away well task collected by ref. ^43^ and the reconfigurable four-room maze by ref. ^44^. We implement these analyses in Matlab 2021a.

In the dataset given in ref. ^43^, to define replay events, we use sharp wave ripples detected in the local field potential as provided by the authors, in combination with the population firing rates. We calculate the population firing rate as a histogram with 1 ms bins of all spikes when the animal’s speed is below 5 cm s^−1^ and then smooth the histogram through a Gaussian kernel with 10 ms s.d. A replay event starts when the population rate exceeds its (nonzero) mean before the ripple and ends when the population firing rate drops below its mean after the ripple. We then repeatedly decode the replay trajectory during the replay event, excluding each neuron that spiked during the event once. We follow the memoryless Bayesian decoding algorithm from ref. ^43^

$$p({\mathrm{pos|spikes}})=F/\mathop{\sum }\limits_{j=1}^{M}F,$$

where

$$F=\left(\mathop{\prod }\limits_{i=1}^{n}{f}_{i}{({\mathrm{pos}})}^{{n}_{i}}\right)\exp \left\{-\tau \mathop{\sum }\limits_{i=1}^{n}{f}_{i}({\mathrm{pos}})\right\},$$

where pos is the animal’s position, spikes is the neural spiking with *n*_*i*_ being the number of spikes of neuron *i*, *τ* is the decoding time window, *n* is the number of neurons, *M* is the number of position bins and *f*_*i*_ (*x*) is the position tuning curve (or the rate map) for neuron *i*. We calculate this rate map by discretizing the arena in 2 cm × 2 cm bins, selecting only times when the animal moves faster than 5 cm s^−1^, then counting the amount of time the animal spends in each bin and the number of spikes of neuron *i* in each bin. We smooth both resulting maps with a Gaussian kernel with an s.d. of 4 cm and then divide the latter by the former to obtain a rate map. We verify our decoding accuracy by calculating rate maps from the first half of a session, then decoding position in 250 ms windows throughout the second half of the session and comparing the decoded position to the true position (only including windows when the animal moves faster than 5 cm s^−1^) for all animals for both sessions (Supplementary Fig. 10). To decode replay trajectories, we apply the same algorithm within a 20 ms window that we slide through the replay event by 5 ms steps. To interpolate the location of a spike of the excluded neuron, we find where the spike time falls between decoding window centers and interpolate the spike’s position between those windows’ decoded positions accordingly. We calculate the rate map for the neuron before the replay event and after the replay event and then subtract before from after to get the rate map change. We translate this change map to place the interpolated spike’s location at the origin. The final product is a replay spike-centered change map for a particular spike of a particular neuron for a particular replay event.

We compare these replay spike-centered change maps between replays from home and replays elsewhere. A home replay meets the following three criteria: the animal is within 10 cm of the home well at the time of the replay, the decoded replay position is within 30 cm from the home well at least once and the mean decoded position is at least 40 cm away from the animal’s location. These criteria make sure the replay trajectory passes by but also extends out from the home well, where the animal is physically located. A replay elsewhere meets the following three different criteria: the animal is more than 10 cm away from the home well, the decoded trajectory stays at least 30 cm away from the home well at any time and the mean decoded position is at least 40 cm away from the animal’s location. These replays elsewhere function as a control condition for any results we find for the replays from home because we do not expect replays elsewhere to change the hippocampal map like the replays from home. However, there is a possibility that the replays elsewhere generally occur at different times in the experiment (for example, most home replay may occur early and most replays elsewhere late). In that case, the control does not work—any results will be confounded by time. Therefore, we sample a selection of the replays elsewhere that match the home replays for when the replay event occurs in the experiment. To do so, we calculate a 5-bin histogram of the event time of the home replays, sample an equal number of replay events from the replay elsewhere and bin them the same way and calculate the correlation between the two five-dimensional vectors of histogram counts. If the histogram counts of the home replays and the subsample of replays elsewhere are correlated, this means that the distribution of replay times is similar between the two. We therefore generate 10,000 samples of replays elsewhere and keep the sample with the highest correlation. Given these three groups of replay events (home, elsewhere and matched), we calculate how the replays change rate maps. To do that, we average the replay spike aligned change (calculated as explained in the previous paragraph) for all cells that participate in the events—if a cell has multiple spikes in one replay event, we average the change map for that cell first (Fig. 4h). Additionally, we calculate the average change within an 8 cm radius for each cell in each replay of the three replay groups (Fig. 4i, left) and the average change in 50 steps of 2 cm along 20 uniformly distributed angles (Fig. 4i, right).

In the same dataset, we look for change maps that are anticorrelated with respect to the current home well versus the home well on the day before, because anticorrelation would mean that the cell generalizes its vector relationship to the home well across home well locations (Fig. 5). We average the change maps across all replay events for each cell, then translate this change map so the current home well ends up in the origin, and translate a copy of the change map so the previous home well is at the origin, then correlate the two. We calculate the distribution of these correlations (Fig. 5g) and plot them on the *x* axis (Fig. 5h–j) against replay measurements on the *y* axis. The first of these replay measurements (Fig. 5h) is the replay-change map overlap. If the interpolated locations of replay spikes of a neuron overlap with the location of its rate map changes, it suggests that replay has a role in making the changes happen. We quantify this replay-change map overlap by calculating the absolute of the total change map and multiplying it elementwise by a map of interpolated replay spike locations, smoothed with a Gaussian kernel of 4 cm s.d., then averaging the resulting map. A high overlap can be caused both by a high number of replay spikes or by precise alignment of replay and change. To calculate the contribution of the former, we simply count the number of home replays that the cell participated in (Fig. 5i). To calculate the contribution of the latter, we correlate the absolute change map and the map of replay locations rather than multiplying the two (as in the overlap), to isolate the spike location from the absolute number of spikes (Fig. 5j).

In the dataset of ref. ^45^, we cannot decode replay or detect sharp wave ripples in the local field potential. The number of simultaneously recorded neurons is too small for accurate decoding, and the quality of the local field potential signal is not sufficient for reliable sharp wave ripple detection. Instead, we define replays through ‘nonlocal door spikes’. We look for these nonlocal door spikes in the third of the five sessions that make up a full experiment—two sessions with all doors open, two sessions with certain doors closed and another session with all doors open. The third session is the one in which the animal first discovers which doors have closed. We define a nonlocal door spike as a spike that meets the following three criteria: (1) the animal must be within 10 cm of a closed door, (2) moving slower than 5 cm s^−1^ (3) and the cell cannot have a place field at the animal’s current location. We use the place fields that the authors detected in the original study, from both the second session (doors open, before animal discovers closed doors) and the fourth session (doors closed, after animal discovers closed doors), for the third criterion. We use the same place fields to determine if any new fields occurred after the door closed. We then plot spikes and behavior for two example cells before the first spike in a new field and after the first spike in a new field (Supplementary Fig. 8c), with nonlocal door spikes before the new field spike marked with crosses. We then regress a binary variable that indicates if a cell obtained any new place fields on a binary variable that measures if the cell replayed (that is, had any nonlocal door spikes), with an additional regressor of the cell’s average firing rate (Supplementary Fig. 8d).

### Home-well mapping simulations

To better understand the origin of the neural responses and representations that we discover in the home-away well paradigm of^43^, we simulate them through our model of compositional map-making. We generate a population of synthetic conjunctive hippocampal neurons. In this population, we simulate replays that build a home well map to demonstrate the source and magnitude of replay spike-aligned rate map changes (Fig. 4). We then compare simulated rate maps before and after the home well change on day 2 to show how anticorrelated rate map changes around the previous versus the new home well arise from compositional representations (Fig. 5).

The simulated neural population consists of 10 entorhinal grid cells, 20 entorhinal object-vector cells and 40 entorhinal cells that code for sensory observations. These entorhinal cells give rise to a population of 600 conjunctive hippocampal neurons—10 × 20 = 200 landmark cells (Supplementary Fig. 11a,b), from the conjunction of 10 grid cells and 20 object-vector cells, and 10 × 40 = 400 place cells (Supplementary Fig. 11c), from the conjunction of 10 grid cells and 40 sensory neurons. We simulate the rate maps for each of these neurons as follows. For each grid cell, we define grid peak locations on a triangular lattice with 120 cm between peaks, rotated by 6 degrees, and add a random lattice translation per grid cell. We then calculate grid cell rate maps by centering a 2D multivariate Gaussian with a 2 cm diagonal covariance matrix on each peak location. For each object-vector cell, we sample a uniform random direction and a uniform random distance between 0 cm and 200 cm to obtain the cell’s vector relation to the home well and then place a 2D multivariate Gaussian with a 1.2 cm diagonal covariance matrix at that vector from the home well. As the home well is in a different location on day 1 versus day 2 of the experiment, we generate two sets of rate maps for the object-vector cells, one for each day. We define each sensory neuron by sampling a uniform random 2D coordinate within the arena and placing a 2D multivariate Gaussian with a 2 cm diagonal covariance matrix at that location. Finally, we scale the rate maps of grid cells, object-vector cells and sensory cells by a factor of 20 Hz divided by the grid/object-vector cell/sensory maximum firing rate, so that the maximum firing rate across the population of each of those will be 20 Hz. Having generated all the required entorhinal representations, we now simulate the resulting hippocampal conjunction rate maps simply by calculating the elementwise product of entorhinal grid cell rate maps and object-vector cell rate maps (for hippocampal landmark cells) and entorhinal grid cell rate maps and sensory cell rate maps (for hippocampal place cells). We also scale these conjunctions so they have a maximum firing rate of 20 Hz across the population. Because we have two sets of rate maps for object vector cells, due to the change in home well location on day 2, we also have two sets of rate maps for landmark cells.

With these synthetic neural populations in place, we can now turn to simulate the rate map changes induced by replay. Our model predicts landmark cells that appear in replay before they appear in physical navigation—in other words, their first spike happens in a replay event. To simulate that, we sample replay trajectories and sample spikes from our synthetic hippocampal population along those trajectories. If the replay trajectory passes through the receptive field of a landmark cell for the first time, that landmark will spike, and it will have a firing field at that location for the remainder of the session. Therefore, if we calculate the change in rate map before the replay event (no spikes) versus after the replay event (lots of spikes) for that landmark cell, we will find a large new peak (Supplementary Fig. 12a). The same replay trajectory may also pass through the receptive field of place cells, and these will also spike during replay, but because their representation remains stable (the sensory properties of the environment do not change), the corresponding rate map changes will be small (Supplementary Fig. 12a). According to our model, we particularly expect replays from home to lay down landmark memories. We therefore contrast such replays with replays elsewhere. In this simulation, replays elsewhere may also elicit place cell and landmark cell spikes, but they will never create new landmark cells—so a landmark cell will never fire for the first time in a replay elsewhere (Supplementary Fig. 12b). This means that toward the end of the session, when most of the landmark cells have already been created in previous home replays, the difference between home and away replays diminishes (Supplementary Fig. 12c,d). Moreover, because these landmark cells have been around for a while, the difference in their rate map before versus after the replay will be small. Therefore, when we plot the average rate map change around replay spikes (Fig. 4f) and average this change within 8 cm of the replay spike (Fig. 4g), we observe that (1) the map changes around both home replays and replays elsewhere (because both sample landmark spikes), (2) this change is larger for home replays (because only these sample landmark spikes for the first time, which is accompanied by the largest rate map change) and (3) this change is much smaller than the magnitude of the new landmark firing field because it is averaged across the hippocampal population (which includes many place fields that do not change) and across replays (for which rate map changes diminish over the course of the session).

We will now provide the implementation details of the replay simulation described above. To obtain replay spike-aligned rate map changes, we need to simulate (1) replay trajectories, (2) spikes of cells along these trajectories and (3) rate map changes for those cells. We sample 100 replay trajectories, with one home replay followed by three replays elsewhere to roughly match the home/elsewhere ratio in the data. We distribute these replays evenly through time between 10 s and 3,000 s (the length of a recording session). Each replay trajectory starts from the home location (home replays) or a random location in the arena (replays elsewhere) and extends randomly between 100 cm and 300 cm in a random direction, divided over 20 steps. The trajectory is cut short if it leaves the arena or if it approaches the home well within 30 cm for replays elsewhere (to match the analysis of empirical data). That gives us our replay trajectories (step 1). We then sample spikes for each simulated cell from a Poisson distribution where the rate is given by the simulated rate map at the replayed locations. Because a replay step takes 5 ms (as in the data) and the simulated rate maps are modeled after those during awake behavior, the effective firing rate in a replay event is much higher than during behavior. To simulate this, we scale up the firing rates by a factor of 25 to get around 30 replay spikes per replay trajectory, roughly matching the data. Furthermore, we assume that a landmark cell can only spike in a replay elsewhere if it has already spiked at least once in a replay from home. That gives us our replay spikes (step 2). The change in rate map for a landmark cell in a replay at time *t*_1_, after it spiked for the first time at *t*_0_, is given by *t*_0_/*t*_1_ times the total rate map change (that is, the full new landmark peak). This implements the fact that for late replays, the landmark field already existed before that replay, so the change before versus after that replay is smaller (but for the first replay, when *t*_1_ = *t*_0_, it is the full new landmark peak). On top of that, we sample change map noise from a standard normal distribution, multiply by 0.5 Hz, and smooth this with a 4 cm Gaussian kernel. Place cell rate map changes consist of only this noise. That gives us rate map changes for each replay spike (step 3). With these ingredients in hand, we calculate the replay spike-aligned rate map change in the same way as we did for the empirical data.

The neural recordings in ref. ^43^ did not allow for tracking the same cells over multiple days, so to find signatures of compositional vector responses, the analysis of Fig. 5 relies on the total rate map change on the second day. In our simulated neural responses, we have the luxury that we can model the same conjunctions across 2 days to show that compositional conjunctive representations indeed produce the same signature as found in the empirical data. We calculate the cumulative rate map change on day 2 for each hippocampal cell by subtracting the simulated rate maps on the first day from the simulated rate map on the second day and adding change noise as in the replay simulation (Supplementary Fig. 11d). We then calculate the correlation between each cell’s change map aligned on the new home well and the same change map aligned on the previous home well (Fig. 5d), exactly like in the empirical data analysis. We plot the distribution of correlation coefficients across the population of synthetic hippocampal neurons (Fig. 5e, left), but now we know which of these are landmark cells and which are place cells, so we can separate their contributions (Fig. 5e, right).

### Reporting summary

Further information on research design is available in the Nature Portfolio Reporting Summary linked to this article.

## Online content

Any methods, additional references, Nature Portfolio reporting summaries, source data, extended data, supplementary information, acknowledgements, peer review information; details of author contributions and competing interests; and statements of data and code availability are available at 10.1038/s41593-025-01908-3.

## Supplementary information

Supplementary Information Supplementary Note and Supplementary Figs. 1–12.

Reporting Summary

Supplementary Data Supporting data for Supplementary Figs. 7–9.

## Source data

Source Data Fig. 4 Source data.

Source Data Fig. 5 Source data.

## Data Availability

All new data in this study (Figs. 1–3 and Supplementary Figs. 1 and 2) were obtained through simulation, for which the code is made available at https://github.com/jbakermans/state-space-composition. Additionally, this study re-analyzed existing data collected for publication in refs. ^43^ and ^44^. The dataset of ref. ^43^ (Figs. 4 and 5 and Supplementary Figs. 7 and 10) was provided on request by the authors. The dataset of ref. ^44^ (Supplementary Figs. 8 and 9) is publicly available as an online resource under doi 10.25493/7NJQ-ANH at https://search.kg.ebrains.eu/instances/Dataset/6ba32f59-e7a0-4cc3-a465-86e1fdf2ffc9. Source data are provided with this paper.

## Code availability

Code for models, simulations and analyses is available at https://github.com/jbakermans/state-space-composition.
