# Supplementary material for: Constructing future behavior in the hippocampal formation through composition and replay
Source: Nat Neurosci. 2025 Mar 10;28(5):1061–72. doi: 10.1038/s41593-025-01908-3 (PMC12081289; doi:10.1038/s41593-025-01908-3)
Supplement: Supplementary file 1 — Supplementary Note and Supplementary Figs. 1–12. [file 41593_2025_1908_MOESM1_ESM.pdf]

nature neuroscience

Supplementary information https://doi.org/10.1038/s41593-025-01908-3

Constructing future behavior in the hippocampal formation through composition and replay

In the format provided by the authors and unedited

Supplementary Material

for

Constructing future behaviour in the hippocampal formation through composition and replay

1 Optimal replay creates memories where they matter most

Requiring as few replays as possible for accurate behaviour is beneficial as replay takes time, and time is often needed for online behaviour [1, 2]. Making the best use of each replay means prioritising certain replay trajectories over others [3]. Intuitively, the highest priority replays are those that improve behaviour the most. This intuition has been formalised in the RL framework, where replay explicitly assigns credit to states through value backups, to successfully explain a wealth of empirically observed patterns of replay [4]. Constructive replay performs implicit credit assignment by binding object-centric representations, that come with pre-learned policies, to allocentric coordinates. Nevertheless, optimal constructive replay trajectories are qualitatively consistent with those predicted by the best value backups - albeit with a very different neural implementation.

Here, we define optimal constructive replay as making *those* memories that are most impactful for shortening paths towards rewards from anywhere in the environment. Such replay forms new vector representation memories, or consolidates existing ones, at locations where these representations change the policy (for example by providing a reward vector) and are frequently retrieved (for example on common paths to reward). We find optimal replays by enumerating replay trajectories and evaluating how much the resulting memories decrease the expected distance towards the goal, averaged across the environment.

Optimal constructive replays play out along trajectories similar to backups that maximally increase future reward, because both aim to achieve the same goal: a map that supports selecting (ultimately) rewarding actions. Updates to the map take place in locations that have the greatest impact on the overall policy, regardless of the representation that underlies that policy. Upon reward discovery, such optimal updates distribute the reward information back through the environment (Fig 1a), whether through value backups or constructive replays. Once complete, the reward map can be further improved by consolidating the existing memories: additional constructive replays suppress path integration errors, along similar trajectories as 'need'-driven value backups [4]

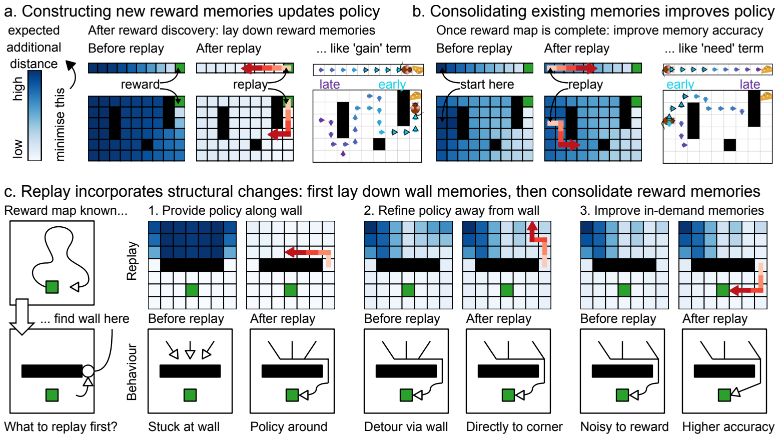

Figure 1: **Optimal replay constructs then consolidates maps.** a. Expected additional distance (blue) for each state before (left) and after (right) the optimal replay (red) upon reward discovery. Optimal constructive replay minimises the total expected additional distance (the sum of additional distance per state plotted in blue). Upon finding a reward, that means laying down reward memories, producing simulated trajectories similar to 'gain'-dominated value backups [4]. b. Expected additional distance (blue) for each state before (left) and after (right) the optimal replay (red) after building the reward map. Once the reward map is sufficiently complete, optimal replays consolidate existing memories that will be needed often in the future, by encoding additional memories to error-correct path integration noise. Such simulated replays play out along similar trajectories as 'need'-dominated value backups [4]. c. Expected additional distance (blue) for each state before (left) and after (right) subsequent (first, second, and third) optimal replays (red) after obstacle discovery. Constructive replay naturally accounts for structural changes, such as the discovery of a wall in the environment. Optimal replay encodes memories where they matter most. In this situation, that produces simulated replays first along the wall to guide the policy around the wall, then away from the wall for a more direct policy, and then towards reward to improve the accuracy of memories that will be retrieved many times.

(Fig 1b). These examples illustrate that the principle of replaying whatever improves the policy most can be applied irrespective of the replay mechanism: trajectories compatible with optimal value backups are often compatible with optimal state-space construction too.

However, that replay mechanism does sharply distinguish the two. Value backups treat the map, whether in hippocampus or cortex, as static, and change striatal synapses to reflect state values. Constructive replays change the hippocampal map itself. A new (replayed) composition that combines a particular vector and grid code recruits a new hippocampal representation - the conjunction between the two. That means that, in addition to reward changes, constructive replay also naturally accounts for structural changes (Fig 1c). We simulate replays when an agent discovers a new wall in a familiar environment with a rewarded location. Optimal replay first runs along the wall (opposite side to the reward) while encoding wall-vector memories. Now all paths behind the wall are successfully rerouted around the wall, though they still go directly to the wall first, and then around. To make these trajectories less bendy, and more efficient, the next replay updates state-representations progressively further away from the wall, so that paths end up going directly to the edge of the wall. Finally, optimal replay consolidates the reward memories that are retrieved most often: those on the path from wall to reward. While no recordings of replay events exist in such situations, exactly these progressively less bendy behavioural trajectories are observed in behaviour when barriers are placed between a start and goal (home) location in a homing task [5].

1.1 Methods

Given the proposed constructive function of replay, what should its content be (Fig 1)? We define a replay as optimal if it maximally reduces the expected additional distance to reward (as compared to before replay), summed across all locations. We search over all possible replays to find the replay trajectory that best improves this metric. Conceptually, there are two ways replay can improve the metric: Either by adding vector representations of previously missing elements into the compositional map, or by consolidating already existing representations to reduce path integration noise. We now describe how we calculate the expected additional distance, and show how replay can improve it by these two ways.

To fully elucidate how the memories formed in constructive replay improve expected additional distance, we need to think about the possible representations at each location. In particular, each location either has access to the full state representation, composed of vector representations for all objects/walls/rewards, or a partial state representation, where an element of the composition is missing - but replay can provide the missing element. Partial representations can have different consequences for policy optimality. Sometimes the resulting policy is still successful (for example, when the missing element is irrelevant for the policy, like a wall to the north when there is reward to the south), but other times the agent will get stuck. Replay must then choose which locations to visit

to make partial state representations full or to further improve representations that are currently noisy.

There are multiple possible ways to formalise expected additional distance. We formalise it by comparing the optimal path versus the path taken by an agent that operates in three regimes related to whether it has access to full or partial representations. The first regime is when it has a full representation - in which case it can path integrate straight to the goal. The second regime is when it has a partial representation - then it can path integrate but may get stuck in states. The third regime is random behaviour - it can switch to this behaviour after it gets stuck. This means the agent will always eventually find the reward. The agent can start using a partial regime then transition into the full regime on visiting a state with a memory of the full state representation, or it can transition from partial to random if the agent gets stuck. We formally model the above using three absorbing Markov chains on the discrete environment. The dynamics of these Markov chain are specified by three transition matrices, defined by policies as $T_{ss'} = p(s'|s) = \Sigma_a p(a|s)p(s'|s,a)$: the transitions T^{full} for the policy given the full state representation, T^{part} for the policy given a partial state representation, and T^{rand} for the random exploration policy. The reward is an absorbing state for each of the chains. For T^{part} there are two additional types of absorbing state: locations where the partial policy gets stuck, for example when there is an unexpected wall in front of the reward, and locations where memories of the full state representation have been encoded. Together, these three chains allow us to analyse a process where an agent that does not have access to the full state representation follows the partial policy until it arrives at reward, arrives at a memory, or gets stuck. If it arrives at reward, it is done (1). If it arrives at a full representation memory, it switches to the dynamics of the full policy T^{full} that takes it to reward (2). If it gets stuck, it starts random exploration following T^{rand} until it either arrives at reward, or hits a memory that provides it with the full representation, so it can follow T^{full} to reward (3). The total expected distance for a location to reward thus becomes the sum of the expected distances of all these scenarios, weighted by their probabilities:

$$
\begin{aligned}
d_{start \to reward} = & \\
1) \quad & p^{part}(start \to reward) d^{part} \\
2) \quad & + \Sigma_{mem} p^{part}(start \to mem)(d^{part}_{start \to mem} + d^{full}_{mem \to reward}) \\
3) \quad & + \Sigma_{stuck} p^{part}(start \to stuck)(d^{part}_{start \to stuck} + p^{rand}_{stuck \to reward} d^{rand}_{stuck \to reward} \\
& + \Sigma_{mem} p^{rand}_{stuck \to mem}[d^{rand}_{stuck \to mem} + d^{full}_{mem \to reward}])
\end{aligned}
$$

To evaluate this expected distance we need to calculate the absorption probability and expected time until absorption for a given chain and a given absorbing state. To get these, standard Markov chain analysis separates the transition matrix T into the dynamics between transient (non-absorbing) states Q and transitions from transient to absorbing states R, and defines fundamental matrix $N = (I_t - Q)^{-1}$, where I_t is the identity matrix with dimension number-of-

transient-states. The probability of getting absorbed at state j, starting from state i, is given by the (i, j)-element of $B = NR$. The expected time until absorption anywhere starting from state i is given by the sum of the i-th row of N. To get the expected time until absorption at *a specific absorbing state* k, we calculate the sum across columns of the fundamental matrix M of an adjusted transition matrix U that expresses transition probabilities given eventual absorption at k: $U_{ij} = B_{ik}T_{ij}/B_{jk}$ [6]. The above considers replay that provides missing elements to the compositional map in memory. But because memories are noisy, due to path integration errors, replay can also improve the policy by encoding memories for elements where these already exist. Repeated replay of existing memories improves the accuracy of those memories. To model this, we additionally inject noise into the transition matrices T^{full} and T^{part}. Because this noise is caused by path integration errors, we model it by mixing the policy at one location with the policy of the neighbours, plus a fixed amount of random policy: $\pi' = m\pi_{noise} + (1 - m)\pi$. Here π' is a vector of action probabilities, π is the noise-free policy vector, and π_{noise} is the mean of the policies across all neighbours and a random policy with equal probability for each action. The amount of mixing m, which determines the policy noise level, is lowered from 0.2 to 0.1 when replay creates an additional memory of an existing representation at that location (i.e. goes from 1 memory to 2 memories).

To actually calculate the optimal replay trajectories, we first calculate the expected distance to reward for each location, subtract the true distance to reward, then sum across all locations. Then we sample all possible 5-step replay sequences starting from the agent's current location and recalculate this total expected additional distance given the memories created in that replay. The optimal replay is the one that decreases the total expected additional distance the most.

2 Composing non-spatial and hierarchical building blocks

Constructive replay thus carries structural information beyond just reward to compose representations that imply actions in remote locations. Importantly, this works equally well for non-spatial building blocks. That is important because empirically, hippocampus supports non-spatial reasoning [7, 8], for example through representation of hierarchy [9, 10] and context [11]. Computationally, non-spatial composition opens up a whole range of new problems to which the same principles can be applied. These problems need to a) decompose into building blocks so a common function $f([z^1, z^2, z^3, ...]) = a$ maps states to optimal actions, and b) allow independent forward models for each building block $z^i_{t+1} = g(z^i_t, a_t)$ so building blocks can be replayed separately and then recombined in memory. Path integration is a specific spatial example of such forward models; more generally, they can be learned to capture a broad variety of environment dynamics.

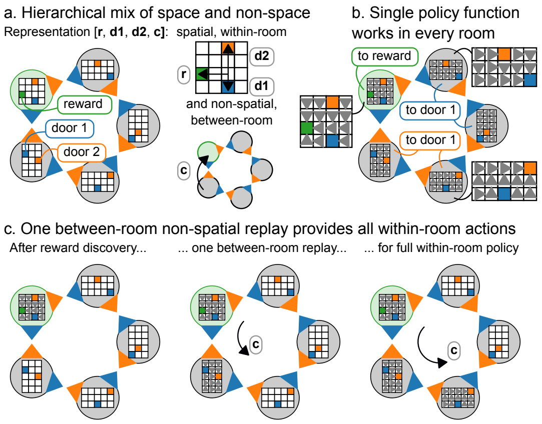

Figure 2: **Policy and replay over hierarchical non-spatial building blocks.** a. An example hierarchical environment that mixes space and non-space, consisting of rooms on a non-spatial loop. One room contains reward (green); each room contains two doors (blue and orange) at random locations that transition the agent to the next room along the non-spatial loop. We provide a full state representation that consists of within-room spatial vectors to reward and doors (r,d^1,d^2) and a between-room non-spatial vector code (c) to the rewarding room. b. Learned policy (grey arrows) in a new hierarchical environment from a policy mapping trained on hierarchical compositional state representations. A policy mapping $f([d^1, d^2, r, c]) = a$ learned across many hierarchical environments provides optimal actions in unseen new configurations, using the same within-room vector codes (d^1,d^2,r) in each room. c. Upon finding a reward, and replaying its within-room location, a single between-room replay propagates the rewarding room-vector code, c, thus providing the optimal policy to each of the other rooms.

We now show that our model works in a hierarchical task that mixes space and non-space. This task consists of multiple spatial rooms (one of which contains a reward) joined together into a (non-spatial) loop by doors, but where the doors are at random spatial locations within each room and behave like teleports (i.e. the locations of the doors are unrelated to the rooms they transition to; Fig 2a). We provide a state representation that composes both spatial within-room vector codes, towards doors (d^1,d^2) or reward (r), and a non-spatial between-room vector code, that specifies the number of rooms between the current and the rewarding room (c). Importantly the d^1, d^2 and r representations are reused across different rooms. As before we train a neural network $f([d^1, d^2, r, c])$ to predict optimal actions on tasks with many different room sizes and door locations, and test on an entirely new hierarchical environment. We see that the network generalises to unseen configurations and provides accurate policies in each room towards the rewarded room (Fig 2b). This highlights generalisation across the hierarchy as anything learned in one room applies in another; alternative approaches like hierarchical RL would need to learn separate policies (or options) in each room.

In the situation described above, we provided the state representation. An agent, however, can only initialise a reward vector code (or rewarding room code) after actually observing a reward in one of the rooms. Similarly to before, we use replay to propagate this knowledge within the rewarded room (i.e. replay r vectors), but additionally replay across rooms (via c). This replay is thus decoupled over space (r) and non-space (c vectors). Since the replay of c is at a hierarchical level, a single replay suffices to obtain the optimal policy in all unrewarded rooms (Fig 2c). The non-spatial between-room vector c can be viewed as a contextual signal that modulates behaviour depending on the state of the environment (like chopping or frying, depending on the current stage of a recipe). And because its forward model can implement arbitrary non-spatial dynamics (such as the non-spatial loop), it could guide actions in game environments that are currently challenging for AI agents [12].

2.1 Methods

Here we show how our model of state-space composition can be extended to accommodate hierarchies of spatial and non-spatial structures (Fig 2). As an example of such an environment, we consider five discrete spatial regular square grid rooms, with shapes that are randomly sampled from (4×4, 3×5, 5×3), connected on a length-5 non-spatial loop. Now transitions exist on both hierarchical levels, within-room and between-room; in general, there can be many recursive levels where a location on the higher level corresponds to a whole environment on the lower level, and a lower-level action can take the agent to a different higher-level location. Here, the low-level rooms contain two doors, which when entered transition the agent to the adjacent high-level room, and one of the low-level rooms contains reward. Crucially the doors are located randomly within each room so their location tells you nothing about the high level action. We provide the agent a state representation that consists of within-

room door and reward vectors d^1,d^2,r and between-room vector c that specifies the clockwise and anticlockwise distance towards the rewarding room.

Like before, we train a policy mapping $f(s) = a$, where the state representation input concatenates vector representations across hierarchical levels $s = [d^1, d^2, r, c]$ and the action output produces an 'primitive' action, on the lowest level of the hierarchy. Intuitively, it makes sense that this state representation affords correct policies: the c representation tells the agent which door to use, and the d^1,d^2 representations tells them how to get to that door - or directly to reward following r if already in the rewarding room. The agent can therefore reuse the same object vector cell population in every room (Fig 2)b). We implement f as a feedforward neural network with one hidden layer of twice the size of the input dimension. To train f, we generate many different environments where the shapes of the rooms, the door locations, and the rewarding room and reward location are randomised. We train f through supervised learning by backpropagation on 500 samples of [state representation, optimal action] pairs from 25 of such environments in parallel, and repeat this 10 times with new environments.

Since door locations are different for each room, the agent utilises a low-level memory bank for each room that binds within-room vector representations to low-level locations. Additionally, the agent has a high-level memory bank that binds between-room vector representations to high-level rooms. This setup factorises the different levels of the hierarchy, and so replay can be independent for the different levels of the hierarchy.

When the agent has, through latent learning, built low-level memories of all door-vectors d^1,d^2 within every room before finding a reward, it has complete maps of the low-level environments but no optimal policy yet - it knows where the doors are, but not yet which door to take. But when it finds the reward in the rewarding room, it only needs to replay at the high-level to create a memory that binds the between-room reward vector c to the room to get access to the optimal policy (Fig 2)c). Next time when it enters a room, it retrieves c from high-level memory and d^1,d^2 from the low-level memory bank for that room, which tells it both where the doors are and which door to approach.

3 Model implementation details

3.1 Parameters

List of parameters with default values. These values are not fixed across simulations: often a simulation systematically varies one or more of these parameters (e.g. the path integration noise, or the number of replays).

Name	Value	Description
l_d	7	Number of nodes along one side of square grid graph
n_b	25	Number of training samples in batch, each from a different environment
n_s	200	Number of training samples from the same environment
n_e	20	Number of times new training environments are generated
ϵ	0	Path integration error probability
w	0.2	Weight of path integrated representation (versus memory retrieved representation) in representation update
r_n	5	Number of replays repeats at one step
r_l	15	Replay length: number of steps in one replay
r_f	4	Replay interval: number of physical steps after which to replay
l_c	1	Length of continuous two-dimensional arena side
n_c	100	Number of object vector cells in object vector representation of one object
d_R	0.1	Maximum step size
σ_r	0.01	Step size variation standard deviation
σ_{theta}	$0.1 \cdot 2\pi$	Step direction variation standard deviation
l_w	0.025	Wall radius (or half the wall thickness)
r_o	0.05	Radius for perceiving objects
ϵ_r	0.025	Path integration step size noise standard deviation
ϵ_θ	$0.25 \cdot 2\pi$	Path integration step direction noise standard deviation
ξ_M	0.05	Distance cutoff for including memories in retrieval
β_M	1	Memory retrieval weight softmax inverse temperature

3.2 State representation

3.2.1 Discrete model

The state space of the discrete model is defined on a set of states $s \in \mathcal{S}$, connected by actions $a \in \mathcal{A}$, to form a graph as in a deterministic Markov decision process. All spatial environments are regular square grid graphs, with actions 'go-north', 'go-east', 'go-south', and 'go-west'. We generate environments by populating a $l_d \times l_d$ square grid with walls and rewards: we assign nodes a 'wall' or 'reward' identity. Wall nodes can be horizontal or vertical neighbours and cannot overlap with rewards or completely block off parts of the environment. We disconnect wall nodes from their non-wall neighbours in the environment by disabling actions that would transition the agent to the wall node.

The full compositional state representation $\vec{v}$ of state s consists of the concatenation of the vector representations $\vec{v}_k$ across all components k in the environment. We represent a reward as a single component, and a wall as a pair of components, one on each end of the wall. For example, the full representation of a state in an environment with two walls and one reward will be $\vec{v} = [\vec{v}_{w_1 1}, \vec{v}_{w_1 2}, \vec{v}_{w_2 1}, \vec{v}_{w_2 2}, \vec{v}_r]$ where [] means concatenation and $\vec{v}_k$ is a vector

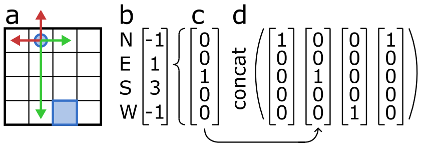

Figure 3: **Discrete object vector representations.** a. Example object vector representation at state s indicated with the blue circle for the blue square reward component at state q. We calculate distances along each action to the component, plotted by green arrows; red arrows indicate actions that go in opposite direction. b. The full object vector representation is a vector of distances along each action, here 'go-north' (N), 'go-east' (E), 'go-south' (S), and 'go-west' (W). Opposite actions are indicated by a -1. c. We one-hot encode each action distance. d. The full state representation $\vec{v}$ that serves as input for f is the concatenation of these one-hot encoded distances.

representation. We implement this vector representation $\vec{v}_k$ as follows (Figure 3). For state s, we get the representation $\vec{v}_k$ of component k at state q as the concatenation of one-hot encoded distances along all actions from s to q (Figure 3a). We set the distance to -1 for an action if q is in the opposite direction of that action (Figure 3b). For example, if q is 1 step to the east and three steps to the south of s, the state representation $\vec{v}_k$ at s becomes $[onehot(-1), onehot(1), onehot(3), onehot(-1)]$ (Figure 3c,d). Here [] means concatenation and $onehot(i)$ is the (i+2)-th column of the $(l_d + 1)$-dimensional identity matrix: each one-hot vector has dimension $l_d + 1$, to enable encoding of all possible distances in the square grid environment, plus the -1 for opposite direction in the first dimension. The resulting $(4\ l_d + 4)$-dimensional vector thus always contains 4 ones, one for each action. Finally, in addition to the vector representations for all components in the environment, we implement a one-hot place code (our model's analogy to the brain's grid code - a coordinate system that uniquely identifies each state) for state s as the i_s-th column of the $(l_d \cdot l_d)$-dimensional identity matrix.

3.2.2 Continuous model

Continuous environments are specified on two-dimensional continuous (x, y) coordinates in an $l_c \times l_c$ arena, so $\vec{s} = \{(x, y)|0 \leq x \leq l_d; 0 \leq y \leq l_d\}$. We place reward and walls within this arena (Figure 4). Again, a reward is represented by a single vector code, and a wall by two vector codes, one centred on each wall end (Figure 4a,b). And like the discrete environment, the full compositional representation for a state concatenates the vector representations for all components, e.g. $\vec{v} = [\vec{v}_{w_1 1}, \vec{v}_{w_1 2}, \vec{v}_{w_2 1}, \vec{v}_{w_2 2}, \vec{v}_r]$. But the individual vector representation $\vec{v}_k$ now consists of the firing rates of a population of n_c spatially tuned object-vector cells (Figure 4c,d). For a component k at state $\vec{q}$, we distribute the firing fields of the object-vector cells evenly on a $2 \cdot l_c \times 2 \cdot l_c$ square grid, with the population's centre aligned on $\vec{q}$. This configuration makes sure that the whole

arena is covered by object-vector cells for any possible $\vec{q}$. We model the firing field of a single object-vector cell as a two-dimensional multivariate Gaussian, with a diagonal covariance matrix that has firing field width Σ on the diagonal and zeros off-diagonal. Then to obtain the vector representation $\vec{v}$ at state $\vec{s}$ for element k, we evaluate the $\vec{q}$-centred population of n_c multivariate Gaussians at $\vec{s}$ to get a n_c-dimensional vector of activities.

Like in the discrete environment, we also implement a place code that represents the absolute location of a state, analogous to grid cells that encode a coordinate system in 2d space to uniquely index location (Figure 4e). In the continuous environment, this place code is obtained by evaluating $n_c/4$ multivariate Gaussians that are arranged at fixed locations on a regular square grid within the arena.

3.2.3 Conjunction versus concatenation

Throughout this paper, state representations are sometimes written as conjunctions and sometimes as concatenations. To avoid confusion, we'll distinguish the two as explicitly as possible here. A concatenation creates a new representation that stacks its component representations; the dimension of the new representation will be the sum of its component dimensions. We use concatenated representations to produce policies from a set of vector representations (e.g. a reward-vector code and a wall-vector code): we map the concatenated vector representations to actions (Section 3.3). A conjunction creates a new representation that combines its component representations, for example through an outer product; the dimension of the new representation will be the product of its component dimensions. We use conjunctions to bind vector representations (e.g. a reward-vector code) to place representations (e.g. a grid code) and store this combination in memory (Section 3.4). Importantly, these conjunctive memories can be created independently for each component: the agent can create a map of grid-reward conjunctions, and a map of grid-wall conjunctions, at different times and different places. Then when they need to select an action at a particular location, encoded by the grid code, they can retrieve the corresponding vector codes, e.g. the reward-vector and wall-vector, concatenate them, and infer appropriate action (Section 3.5). In summary: in our model conjunctions are for memory, and concatenations are for policy.

3.3 Learning policies that generalise

3.3.1 Discrete model

Given the full state representation that concatenates vector codes for all objects in the environment, e.g. $\vec{v} = [\vec{v}_{w_1 1}, \vec{v}_{w_1 2}, \vec{v}_{w_2 1}, \vec{v}_{w_2 2}, \vec{v}_r]$, the model learns a policy through a mapping $f(\vec{v}) = \vec{p}_a$ that maps a state representation to action probabilities. Here $\vec{p}_a$ is a vector of action probabilities, so its dimension matches the number of actions in the environment, i.e. four in the square grid world. The mapping f is learned across many environments, so that it generalises to new

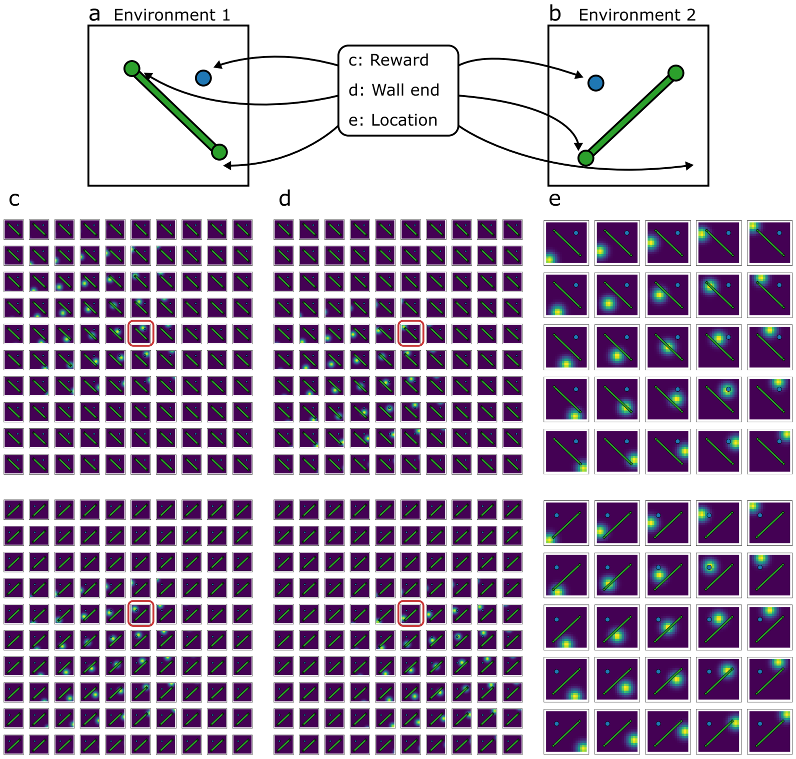

Figure 4: **Continuous object vector representations.** a. Continuous environment with a reward (blue circle) and a wall (green bar), represented by two wall ends (green circles). b. Continuous environment with different configuration. c. Object vector cell population for the reward object. Each square is a rate map for one cell, the top row for the environment in *a* and the bottom row for the environment in *b*. The rate map circled in red, for example, shows a cell that fires just west of the reward in both environments. d. Object vector cell rate maps for one wall end, with the cell circled in red firing just west of the wall end in both environments. e. Place cell population that represents location in both environments, independent of object orientation.

configurations of objects on first encounter. We implement f as a multi-layer perceptron, consisting of an input layer of the same dimension as s (i.e. $(4\ l_d + 4)$*number of components), then three hidden layers of dimensions 1000, 750, 500 and rectified linear activation, and an output layer of the same dimension as a (i.e. 4). Before inputting $\vec{v}$ into f, we preprocess all wall vector representations with a common wall-embedding function f_w, with an output layer of the same dimension as the wall representation and a hidden layer twice that size and rectified linear activation. In the example $\vec{v} = [\vec{v}_{w_1 1}, \vec{v}_{w_1 2}, \vec{v}_{w_2 1}, \vec{v}_{w_2 2}, \vec{v}_r]$, that means that the input to f would be $[f_w(\vec{v}_{w_1 1}, \vec{v}_{w_1 2}), f_w(\vec{v}_{w_2 1}, \vec{v}_{w_2 2}), \vec{v}_r]$. This wall

embedding is not necessary for accurate policies, but does speed up learning.

We train f and f_w through supervised learning on the optimal policy. As training examples, we generate n_b environments with random configurations of walls and rewards, and calculate the representation s and optimal policy a for each state. We use Dijkstra's algorithm to determine shortest-path distances from each location to the reward, while taking walls into account; the optimal policy then assigns equal probability to all actions that decrease the distance to reward, and zero to others. We then sample a random node from each environment and train f on the batched $\{\vec{v}, \vec{p_a}\}$ pairs. We update f's parameters through backpropagation using gradients provided by Pytorch and the ADAM optimiser with learning rate 10^{-3}. Because f is trained on multiple environments in parallel, it needs to learn a mapping that works across environment configurations. After n_s samples we generate new training environments, and this procedure is repeated n_e times.

3.3.2 Continuous model

In continuous environments, we define actions as optimal directions to reward. Therefore mapping $f(\vec{v}) = \vec{\phi}$ must produce a continuous, periodic value $-\pi \leq \theta \leq \pi$. We implement this action output as a two-dimensional vector $\vec{\phi}$ that contains the sine and the cosine of the optimal direction θ; the optimal direction can be recovered from this output as $\theta = atan2(\phi_2, \phi_1)$. Again, the input state $\vec{v}$ concatenates the vector representation across all components in the environment, e.g. $\vec{v} = [\vec{v}_{w_1 1}, \vec{v}_{w_1 2}, \vec{v}_{w_2 1}, \vec{v}_{w_2 2}, \vec{v}_r]$ – with vector representations calculated as described in Section 3.2.2. The network hidden state dimensions are 3000, 2000, 1000, and we preprocess wall embeddings as in the discrete model to speed up learning. As training examples, we generate n_b environments with random configurations of walls and rewards, and sample random locations within those to provide training state-action pairs. We calculate the optimal direction θ for a location as follows. First, we construct a fully connected graph of the environment, where each component in the environment (with two components for a wall, one on each end) is a node, and with edges whose weight is the Euclidian distances between objects. We remove edges between objects if the line connecting them in the environment crosses any walls. Then we use Dijkstra's algorithm to calculate the graph distance to the nearest reward for each node on this graph, taking the edge weights into account. Finally, for a given location $\vec{s}$, the optimal direction points towards the object that a) can be reached in a straight line without crossing walls from $\vec{s}$ and b) has the smallest graph distance to reward after adding the distance from that object to $\vec{s}$. Provided with these state-action training examples, we update f's parameters through backpropagation as in the discrete model. After n_s state-action training examples we sample new training environments, and we repeat this n_e times.

3.4 Mapping the environment

3.4.1 Discrete model

Given a learned mapping $f(\vec{v}) = \vec{p}_a$, the agent can act optimally when it has access to its full state representation $\vec{v}$. However, upon entering a new environment, they don't know the current configuration of components. They will need to incorporate the components in their state representation through exploration (Figure 5). A simulated episode in such a new environment runs as follows. Initially, all vector representations are initialised as empty. The agent explores the environment by observing their location, selecting an action from their exploration policy (e.g. a random policy with equal probability for all available actions), and transitioning to the next location. When they discover a component, by approaching it within one step for wall ends or zero steps for reward, they initialise the object-vector representation $\vec{v}_k$ for that object k. For ease of notation and implementation, we will keep track of the represented state s_k of the agent for that component rather than the vector representation $\vec{v}_k$. This representation can be thought of as "where does the agent think they are, with respect to component k". For example, when the agent discovers a component k at state $q = 12$, and takes a step east, we'll write $s_{k,q=12} = 13$ for their new vector representation of that component instead of $\vec{v}_k = [onehot(0), onehot(-1), onehot(0), onehot(-1)]$ ('1 step west'). That makes the equations easier to read, but the two notations are equivalent, because there is a one-to-one correspondence between represented state and object-vectors (as described in Section 3.2.1). We'll drop the subscript q in the following because for a particular component k, the state it is in, q, is fixed.

After initialisation, the agent carries out such updates to vector representations with respect to their actions through path integration (Figure 5a). However, path integration is noisy. Errors may cause the represented component vector state s_k to diverge from the true state s. We model path integration noise as a distribution over transitioned locations s'_k given action a from state s_k:

$$p_{PI}(s'_k|s_k, a) = \begin{cases} \frac{1-\epsilon}{|S'(s_k,a)|} & \text{if } s'_k \in S'(s_k, a) \\ \frac{\epsilon}{|S''(S'(s_k,a))|} & \text{if } s'_k \in S''(S'(s_k, a)) \\ 0 & \text{otherwise} \end{cases} \tag{1}$$

where

$$S'(s_k, a) = \{s' \in \mathcal{S} \mid p(s'|s_k, a) > 0\} \tag{2}$$

is the set of locations that taking a from s_k can transition to ($p(s'|s, a)$ is defined by the environment; here, since the environment is deterministic, $|s'| = 1$), and

$$S''(S') = \{s'' \in \mathcal{S} \mid s'' \notin S' \text{ and } \sum_{s'}^{S'} \sum_{a'}^{\mathcal{A}} p(s''|s', a') > 0\} \tag{3}$$

is the set of two-step neighbours of s_k. In other words, path integration updates the representation to the correct location s'_k with probability $1 - \epsilon$ or any of

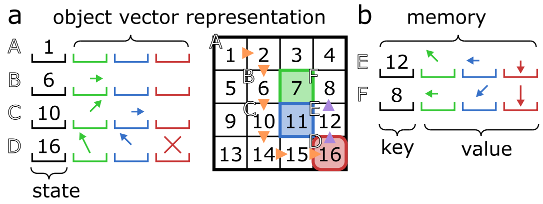

Figure 5: **Discrete map-making.** a. Schematic of a model trajectory after entering a new environment for the first time. During exploration (orange arrows), the model observes states (numbers, black slots) and tracks object vector representations (arrows in green, blue, and red slots) through path integration. Vector representations start empty (step A), but are initialised on component discovery (like the green wall end, step B). As the agent moves from state 6 to state 10, they uses their knowledge about their actions to path integrate the representations of the green wall (so that now in state 10 it points north-east). In addition, the model discovers the blue wall, and initialises its representation. b. At each step, the model stores the vector representations it has access to in memory by binding it to the state, implemented as a dictionary where the state (black) is the key and the representation (green, blue, red) the value. In replay, the model carries state representation to remote locations without physical displacement (purple arrows). It then binds the object vector representations to locations in memory (step E, F) for later retrieval at that location.

the correct location's neighbours with total probability ϵ. Note that this accumulation of error occurs independently for each component k, resulting in a (possibly) different representation of "where do I think I am" with respect to each component.

In addition to path integration, the agent relies on memory to update representations after an action (Figure 5b). In the brain, we hypothesise these memories are stored as hippocampal conjunctions, which link a vector representation (the object-vector cell population activity) to place representations (the grid cell population activity).

Here, we model such conjunctive memories as a dictionary $M_s = [s_k^i, ...]$ with states s as keys and multisets (i.e. the same element can appear multiple times) of vector representations (or, again for ease of notation: the state s_k corresponding to a vector representation) as values. The superscript i indexes all the memories that exist at state s: the agent appends a new memory s_k to the memory bank M_s at state s every time they go there. Without path integration errors, the represented state s_k always matches the true state s, so in that case M_s would just contain many copies of s. But due to path integration errors, the represented state might diverge from the true state, so that the list of memories M_s contains a variety of states s_k that are not s. In the example of taking a step east after finding reward at state 12, the new memory would be $M_{13} = [13]$, indicating a reward one step to the west at state 13 – if they have correctly path integrated their reward-vector representation. If they made a path integration

error, the encoded memory may instead be $M_{13} = [14]$, (incorrectly) indicating a reward two steps to the west. When the agent returns to a location where it has previously encoded memories, they can retrieve these memories. We model the probability of retrieving a particular vector representation s_k as proportional to the number of encoded memories of that vector representation at the current state s:

$$p_M(s_k|s) = \frac{|[s_M \in M_s \mid s_M = s_k]|}{|M_s|} \tag{4}$$

which is only calculated if $|M_s| > 0$, so there are memories at state s. Equation 1 and 4 thus provide two probability distribution of updated vector representations s'_k given the previous representation s_k, the action the agent took a, and the new state s. To update their representation, the agent can make use of the combination of the two: a weighted sum over the path-integrated representation (weight w) and the distribution of representations on earlier encounters of this state (weight $1 - w$), setting $w = 1$ if $|M_s| = 0$.

$$p(s'_k|s_k, a, s) = w p_{PI}(s'_k|s_k, a) + (1 - w) p_M(s'_k|s) \tag{5}$$

They sample s'_k from Equation 5 to obtain the updated representation, which is encoded in memory and path-integrated in the next step.

Replay in our model allows the agent to encode remote memories offline. Given an initial state s and representation s_k, they imagine transitions through the environment. After each transition, they update their representations and append them to the current state's memories, as during online navigation. There is one difference: Since transitions are now imagined, the current state cannot be observed from the environment. Instead, it needs to be path integrated, just like the object-vector representations. That means there can now be errors both in the state s (the key in the memory dictionary) and the vector representation (the value in the memory dictionary). Again, we model path integration noise by sampling from a distribution over correct and neighbour locations. The agent performs r_n replays of r_l steps long, every r_f steps of exploration.

3.4.2 Continuous model

During simulated exploration, the continuous space agent takes steps d of length d_r (capped at d_R) in direction d_θ by adding normal random variation with standard deviation σ_r, σ_θ to the previous length and direction for smooth trajectories. Compared to the discrete environments, where we simply disabled actions that lead into walls, the interaction with continuous walls is a bit more complicated. The agent cannot pass through walls, but should still be able to slide along walls if they are not moving perpendicularly towards the wall, unless there is another wall preventing that (as in corners). Algorithm 1 resolves an attempted step $\vec{d}$ from state $\vec{s}$ to $\vec{s}'$ in the presence of walls, with wall normal vectors pointing from $\vec{s}$ orthogonally into the wall.

The agent thus explores the environment (Figure 6). They discover a component when they get within radius r_o of that component, and initialise the

Algorithm 1 Transition with walls

1. Is $\vec{s}$ within distance l_w of a wall?

Yes Is $\vec{s}$ within distance l_w of multiple walls?

Yes Calculate the dot products of $\vec{d}$ and the normal vectors of the walls within l_w

Remove the positive component along the normal vector of the wall with the largest dot product from $\vec{d}$.

Recalculate the dot product of $\vec{d}$ and the normal vector of the wall that had the second-largest dot product. Is it greater than zero?

Yes Set $\vec{d}$ to $\vec{0}$; *continue to 2.*

No *continue to 2.*

No Remove the positive component along the normal vector of the wall within l_w from $\vec{d}$; *continue to 2.*

No *continue to 2.*

2. Does the line from $\vec{s}$ to $\vec{s}+\vec{d}$ cross any walls?

Yes Set new location $\vec{s}'$ to wall contact location, l_w away from the wall crossed first; *exit.*

No Set new location $\vec{s}'$ to $\vec{s}' = \vec{s}+\vec{d}$; *exit.*

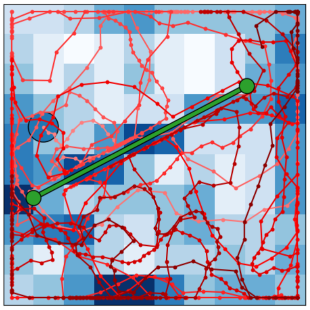

Figure 6: **Continuous exploration.** Example walk of agent in a continuous environment, each step marked with a red dot connected to the previous step with a red line, progressing from light red to dark red over the course of the walk. Blue patches indicate binned location occupancy. Interactions with the green wall and arena boundaries demonstrate how the agent slides along blockades without crossing them.

corresponding vector representation upon discovery. Again, for ease of notation, we will write the agent's represented state $\vec{s}_k$ for an object instead of the object-vector representations $\vec{v}_k$. In other words, we'll write representations as 2-dimensional (x, y) vectors, but these have a one-to-one correspondence with n_c-dimensional population activities of object-vector cells. After initialisation, the agent tracks the object's representation by combining path integration with memory retrieval, just like in the discrete model. Path integration updates represented states $\vec{s}_k$ to reflect step $\vec{d}$, with path integration error modelled as Gaussian noise with standard deviation e_r and e_d on step length d_r and direction d_θ respectively:

$$\begin{aligned}
\vec{s}_k^{PI} &= \vec{s}_k + \vec{d}^{PI} \\
\vec{d}^{PI} &= d_r^{PI}\cos(d_\theta^{PI})\hat{x} + d_r^{PI}\sin(d_\theta^{PI})\hat{y} \\
d_r^{PI} &= d_r + e_r, e_r \sim \mathcal{N}(0, \epsilon_r) \\
d_\theta^{PI} &= d_\theta + e_\theta, e_\theta \sim \mathcal{N}(0, \epsilon_\theta)
\end{aligned} \tag{6}$$

Note that equation 6 follows the state update algorithm, taking walls into account.

We encode a new memory as a state-representation pair, so that the memory bank $M = [\{\vec{s^i}, \vec{s}_k^i\}, ...]$ becomes a multiset of pairs. The reason this is different from the dictionary in the discrete case, is that states are now continuous coordinates, so there is not much point in using them as dictionary keys: it is unlikely the same key will occur twice. Again, this memory bank is a simplified implementation of what we hypothesise the brain achieves through hippocampal conjunction: the binding together of a location and object-vector code. Then to retrieve a representation at state $\vec{s}$ from all previous experience, we calculate a weighted average across representations $\vec{s_k}^i$ in the memory bank. The weights are given by the corresponding memory states $\vec{s^i}$: memories created near $\vec{s}$ contribute stronger to the retrieved representation. More specifically, we exclude memories create beyond distance threshold ξ_M, and then weight remaining memories by the softmax of dot products between the memory locations $\vec{s^i}$ and retrieval location $\vec{s}$:

$$\vec{s}_k^M = \frac{\sum_{\{\vec{s^i}, \vec{s}_k^i\} \in V} e^{\beta_M \vec{p}(\vec{s}) \cdot \vec{p}(\vec{s}_i)} \vec{s}_k^i}{\sum_{\{\vec{s^i}, \vec{s}_k^i\} \in V} e^{\beta_M \vec{p}(\vec{s}) \cdot \vec{p}(\vec{s}_i)}} \tag{7}$$

where β_M is the softmax inverse temperature, $\vec{p}(\vec{s})$ the place code of a given (x, y) location (see Section 3.2.2) and V only contains the included memories:

$$V = [\{\vec{s^i}, \vec{s}_k^i\} \in M \mid |\vec{s} - \vec{s^i}| < \xi_M] \tag{8}$$

The final updated representation combines the path-integrated and retrieved representation as a weighted sum, again setting $w = 1$ if $|V| = 0$:

$$\vec{s}_k = w\vec{s}_k^{PI} + (1 - w)\vec{s}_k^M \tag{9}$$

This final updated representation is encoded in memory and path-integrated in the next step. The replay implementation in the continuous model is very similar to the discrete case. The agent imagines transitions and makes a new memory at each replay step, path-integration both location and representation – in contrast to physical navigation, where the location $\vec{s}$ is observed from the environment.

3.5 Goal-directed navigation

Following the map construction of Section 3.4, the agent can read out the policy given a state representation through the policy mapping learned as described in Section 3.3. By carrying out these actions, the agent engages in goal-directed navigation or exploitation, in contrast to Section 3.4 which described exploration. Practically, we first convert the represented locations to vector codes for each object as described in Section 3.2, and then concatenate the vector codes across objects to obtain the input vector for f. The agent then executes the action (in the continuous model, this means taking a step of size d_R in the direction produced by f), updates their representations, and repeats the procedure until reaching reward.

4 Supplementary figures

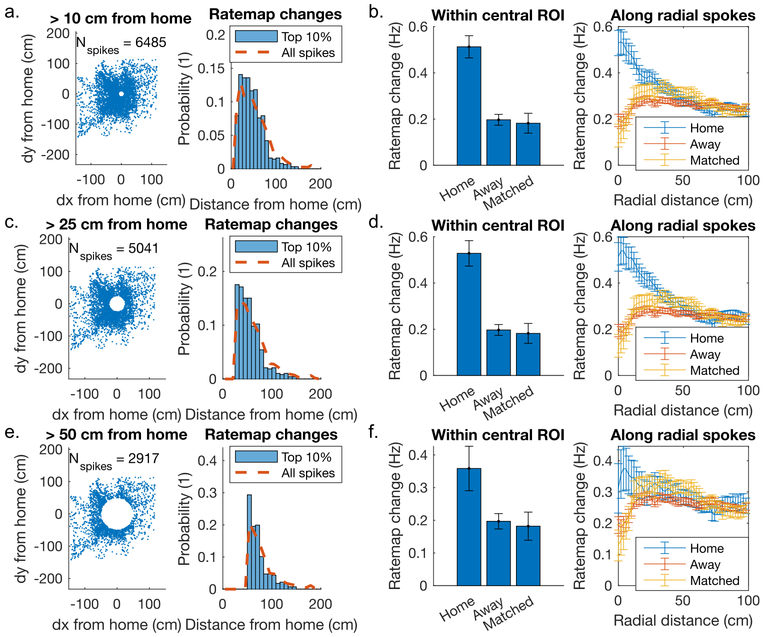

Figure 7: **Contribution of remote replay spikes.** a. To verify that the replay-spike aligned ratemap changes are not just driven by changes at the home-well, we repeat the analysis while excluding replay spikes within 10 cm from the home well. The location of included replay spikes relative to home is plotted on the left, and the distribution of home distances for the top 10 % of replay spikes that change the ratemap the most on the right, showing that many ratemap changes occur at considerable distance from the home well. The baseline distribution of home distances across all replay spikes is shown on top in dashed lines. b. Repeating region-of-interest and radial-spoke averages of ratemap change around replay spikes, only including replay spikes more than 10 cm away from the home well as shown in *a*. N=6485 (home), 20912 (away), 6539 (matched); error bars: s.e.m. c-d. Same procedure, now excluding replay spikes within 25 cm from the home well. N=5041 (home), 20912 (away), 6539 (matched); error bars: s.e.m. e-f. Same procedure, now excluding replay spikes within 50 cm from the home well. As the full arena is 200 by 200 cm, the diameter of the exclusion region now spans half of the arena length. N=2917 (home), 20912 (away), 6539 (matched); error bars: s.e.m. c-d.

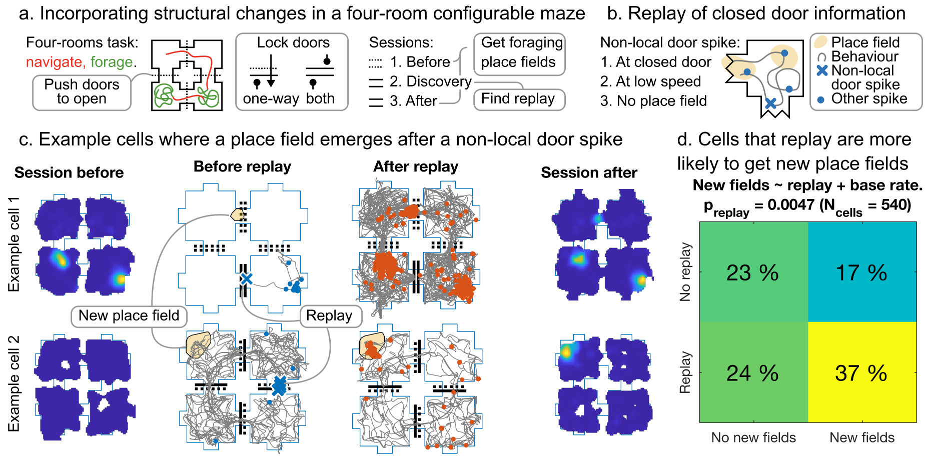

Figure 8: **Replay to incorporate structural elements.** a. We investigate replay of structural changes in a four-room configurable maze task, where the animal needs to navigate to a particular room then forage there. Some doors between rooms close halfway through the experiment; we calculate ratemaps before and after closing and look for replay after discovery of the change. b. Because we cannot detect SWRs or decode replay, we define replay through 'non-local door spikes'. c. Two example cells where such a non-local door spike precedes the emergence of a new place field. d. Across the population, cells with non-local door spikes are more likely to get new place fields. Each recording consists of five sessions: doors open (s1), doors open (s2), doors closed (s3), doors closed (s4), doors open (s5). We find cells that have non-local door spikes when the doors close for the first time (s3) and ask whether the same cells obtain new place fields in the next closed door session (s4) that did not exist in the previous open door session (s2). We regress the emergence of new fields on the presence non-local door spikes, including a regressor for baseline firing rate, and find a significant relation.

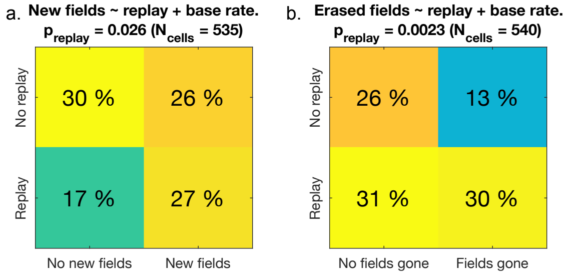

Figure 9: **Replay for stable environments and erasing fields.** a. The relation between replay and new fields also exists in a stable environment, but there is less replay than in a changing environment. We repeat the analysis of Figure 8, but now extract non-local door spikes in session s2 (a stable session), and detect new place field by comparing session s3 to session s1. The relation between the two still holds, but this time there are more cells that have no replay and no new fields (top left) and fewer that have replay and new place fields (bottom right). That is consistent with the model: even in the absence of structural changes, when replay occurs it should still make new landmark cells (to further consolidate the representation). It should just do so less often. This means that the correlation between replay and new cells should still exist, but there should be less replay than in 8, where the environmental structure changes. b. There is also a relation between replay and disappearing fields in a changing environment, but that leaves more replays unexplained. We repeat the same analysis of Figure 8, but focus on disappearing place fields rather then new place fields. We extract non-local door spikes in session s3, but detect place fields that are present in session s2 but not any more in session s4. We find that cells that replay in s3 lose place fields more often, which is consistent with our findings of changes in representations on the second day of the home-away well paradigm. This time the proportion of cells that does replay but does not change firing fields fields (bottom left) is larger than in *a* and 8, which again aligns with our model: we expect many of these replaying cells to gain new place fields too (rather than just lose them), which this analysis does not measure.

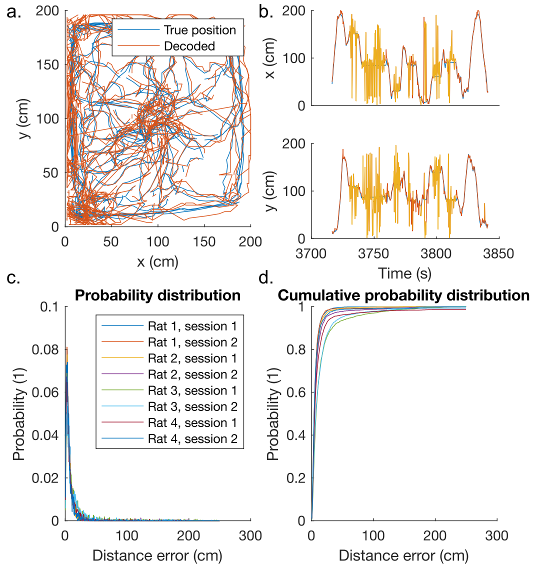

Figure 10: **Decoding accuracy.** a. Decoded trajectory from held-out neural data, plotted on top of the true position of the animal, for an example rat in an example session. This only includes time windows where animal moved faster than 5 cm/s b. Same data as in *a*, but only the first 125 seconds, separated for the x and y position, plotted against time. During periods of immobility (animal speed below 5 cm/s; flat line in true position against time), the decoded position is overlaid in yellow. c. Probability density for decoding error, measured as distance between true and decoded position, across animals and sessions. d. Cumulative probability distribution for data in *c*.

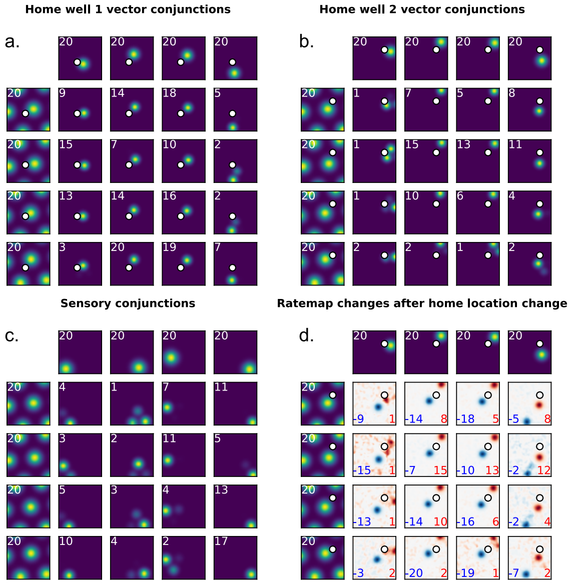

Figure 11: **Simulated ratemaps for home-well experiment.** a. Sample of synthetic entorhinal grid/object-vector cells, and hippocampal landmark cells in the simulation of the home-away well paradigm for the home well location (white circle) of day 1. The first row shows an example subset of 4 object-vector cells (there are 20 in the simulated population), the first column shows an example subset of 4 grid cells (there are 10 in the simulated population), and the remaining rows and columns the resulting 16 conjunctions (landmark cells; there are 200 of those in the simulated population). The white number indicates the max firing rate in Hz. b. Same cells as in *a* but for the home well location (white circle) of day 2. c. Sample of synthetic enthorinal grid/sensory cells, and hippocampal place cells in the same simulation. Like *a* and *b*, except that the top row is now a subset of 4 sensory cells (there are 40 of those in the simulated population), so that the 16 example hippocampal conjunctions (there are 400 in the full simulated population) are now place cells that are stable across days, because the sensory properties of the environment remain identical. d. Ratemap changes for the example subset of landmark cells in *a* and *b* from day 1 to day 2. Positive ratemap changes are plotted in red, negative ratemap changes in blue, with the red and the blue number in the right and the left bottom corner indicating maximum and minimum change respectively.

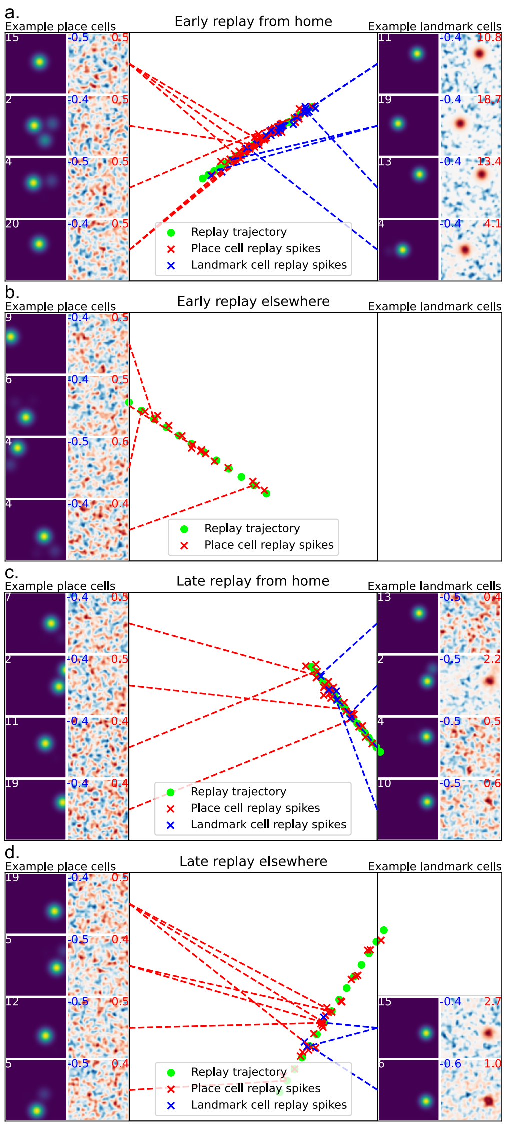

Figure 12: **Example replays in home-well simulation.** a. Example of a simulated replay from home early in the session. The green trajectory plots the replayed trajectory. Red crosses are spikes of place cells during this replay (with small jitter for illustration; else they would all be on top of the green circles, as these are the replayed locations), blue crosses are spikes of landmark cells. (Continued on next page.)

Figure 12: (Continued from previous page.) On the left of the arena there is a random sample of four place cells (left: ratemap, peak firing rate (Hz) in white; right: change in ratemap, min/max change in firing rate (Hz) in blue/red) that fired during this replay; the dashed red lines connect these cells with their replay spikes. There are four random landmark cells that fired on the right of the arena, connected to their spikes with blue dashed lines. Notice large ratemap changes for the landmark cells as these firing fields have just created in this replay. b. Example of simulated early replay elsewhere. Notice that no landmark cells fired in this replay, as their fields have not been created yet. c. Example of simulated late replay from home. Notice the small changes for landmark cells, because their fields have existed for a while, so the ratemap before this replay ends up highly similar to the ratemap after this replay. d. Example of simulated late replay elsewhere. Notice that, in contrast to *b*, there are landmark cell spikes because these landmark cells have been created in previous home replays, but as in *c* the landmark ratemap changes are small.

5 References

References

[1] Mayank Agrawal, Marcelo G. Mattar, Jonathan D. Cohen, and Nathaniel D. Daw. The temporal dynamics of opportunity costs: A normative account of cognitive fatigue and boredom. *Psychological Review*, 129:564–585, 2022.

[2] Kristopher T. Jensen, Guillaume Hennequin, and Marcelo G. Mattar. A recurrent network model of planning explains hippocampal replay and human behavior, January 2023.

[3] Hideyoshi Igata, Yuji Ikegaya, and Takuya Sasaki. Prioritized experience replays on a hippocampal predictive map for learning. *Proceedings of the National Academy of Sciences*, 118(1), January 2021.

[4] Marcelo G. Mattar and Nathaniel D. Daw. Prioritized memory access explains planning and hippocampal replay. *Nature Neuroscience*, 21(11):1609–1617, November 2018.

[5] Philip Shamash, Sarah F. Olesen, Panagiota Iordanidou, Dario Campagner, Nabhojit Banerjee, and Tiago Branco. Mice learn multi-step routes by memorizing subgoal locations. *Nature Neuroscience*, 24(9):1270–1279, September 2021.

[6] David Clyde. Answer to "Expected time till absorption in specific state of a Markov chain", December 2022.

[7] Dmitriy Aronov, Rhino Nevers, and David W. Tank. Mapping of a non-spatial dimension by the hippocampal-entorhinal circuit. *Nature*, 543(7647):719–722, 2017.

[8] J. A. Dusek and H. Eichenbaum. The hippocampus and memory for orderly stimulus relations. *Proceedings of the National Academy of Sciences*, 94(13):7109–7114, 1997.

[9] Kirsten Brun Kjelstrup, Trygve Solstad, Vegard Heimly Brun, Torkel Hafting, Stefan Leutgeb, Menno P. Witter, Edvard I. Moser, and May-Britt Moser. Finite Scale of Spatial Representation in the Hippocampus. *Science*, 321(5885):140–143, July 2008.

[10] Matthew L. Shapiro, Heikki Tanila, and Howard Eichenbaum. Cues that hippocampal place cells encode: Dynamic and hierarchical representation of local and distal stimuli. *Hippocampus*, 7(6):624–642, 1997.

[11] Sam McKenzie, Andrea J. Frank, Nathaniel R. Kinsky, Blake Porter, Pamela D. Rivière, and Howard Eichenbaum. Hippocampal representation of related and opposing memories develop within distinct, hierarchically organized neural schemas. *Neuron*, 83(1):202–215, 2014.

[12] Jane X. Wang, Michael King, Nicolas Porcel, Zeb Kurth-Nelson, Tina Zhu, Charlie Deck, Peter Choy, Mary Cassin, Malcolm Reynolds, Francis Song, Gavin Buttimore, David P. Reichert, Neil Rabinowitz, Loic Matthey, Demis Hassabis, Alexander Lerchner, and Matthew Botvinick. Alchemy: A benchmark and analysis toolkit for meta-reinforcement learning agents, October 2021.
