# Supplementary material for: Constructing future behavior in the hippocampal formation through composition and replay
Source: Nat Neurosci. 2025 Mar 10;28(5):1061–72. doi: 10.1038/s41593-025-01908-3 (PMC12081289; doi:10.1038/s41593-025-01908-3)
Supplement: Supplementary file 2 — Reporting Summary [file 41593_2025_1908_MOESM2_ESM.pdf]

nature portfolio

Corresponding author(s): JJW Bakermans

Last updated by author(s): 4 Dec 2024

Reporting Summary

Nature Portfolio wishes to improve the reproducibility of the work that we publish. This form provides structure for consistency and transparency in reporting. For further information on Nature Portfolio policies, see our Editorial Policies and the Editorial Policy Checklist.

Statistics

For all statistical analyses, confirm that the following items are present in the figure legend, table legend, main text, or Methods section.

n/a	Confirmed	
☐	☒	The exact sample size (n) for each experimental group/condition, given as a discrete number and unit of measurement
☐	☒	A statement on whether measurements were taken from distinct samples or whether the same sample was measured repeatedly
☐	☒	The statistical test(s) used AND whether they are one- or two-sided<br>*Only common tests should be described solely by name; describe more complex techniques in the Methods section.*
☐	☒	A description of all covariates tested
☐	☒	A description of any assumptions or corrections, such as tests of normality and adjustment for multiple comparisons
☐	☒	A full description of the statistical parameters including central tendency (e.g. means) or other basic estimates (e.g. regression coefficient) AND variation (e.g. standard deviation) or associated estimates of uncertainty (e.g. confidence intervals)
☐	☒	For null hypothesis testing, the test statistic (e.g. F, t, r) with confidence intervals, effect sizes, degrees of freedom and P value noted<br>*Give P values as exact values whenever suitable.*
☒	☐	For Bayesian analysis, information on the choice of priors and Markov chain Monte Carlo settings
☒	☐	For hierarchical and complex designs, identification of the appropriate level for tests and full reporting of outcomes
☐	☒	Estimates of effect sizes (e.g. Cohen's d, Pearson's r), indicating how they were calculated

Our web collection on statistics for biologists contains articles on many of the points above.

Software and code

Policy information about availability of computer code

Data collection	All new data were obtained through simulation in Python 3.8.12; neural networks trained using PyTorch 1.10.0 and optimised using the ADAM optimiser. Code is available at https://github.com/jbakermans/state-space-composition.
Data analysis	Simulated data were analysed in Python 3.8.12; neural recordings analysed in Matlab 2021a. Analysis scripts available at https://github.com/jbakermans/state-space-composition.

For manuscripts utilizing custom algorithms or software that are central to the research but not yet described in published literature, software must be made available to editors and reviewers. We strongly encourage code deposition in a community repository (e.g. GitHub). See the Nature Portfolio guidelines for submitting code & software for further information.

Data

Policy information about availability of data

All manuscripts must include a data availability statement. This statement should provide the following information, where applicable:

- Accession codes, unique identifiers, or web links for publicly available datasets
- A description of any restrictions on data availability
- For clinical datasets or third party data, please ensure that the statement adheres to our policy

All new data in this study (Figure 1, 2, 3, S1, S2) were obtained through simulation, for which the code is made available at https://github.com/jbakermans/state-space-composition. Additionally, this study re-analysed existing data, collected for publication in Pfeiffer & Foster (Nature 2013) and Duvelle et al. (Current Biology

2021). The Pfeiffer & Foster dataset (Figure 4, 5, S7, S10) was provided on request by the authors. The Duvelle et al. dataset (Figure S8, S9) is publicly available as an online resource under doi 10.25493/7NJQ-ANH at https://search.kg.ebrains.eu/instances/Dataset/6ba32f59-e7a0-4cc3-a465-86e1fdf2ffc9.

Research involving human participants, their data, or biological material

Policy information about studies with human participants or human data. See also policy information about sex, gender (identity/presentation), and sexual orientation and race, ethnicity and racism.

Reporting on sex and gender	N/A
Reporting on race, ethnicity, or other socially relevant groupings	N/A
Population characteristics	N/A
Recruitment	N/A
Ethics oversight	N/A

Note that full information on the approval of the study protocol must also be provided in the manuscript.

Field-specific reporting

Please select the one below that is the best fit for your research. If you are not sure, read the appropriate sections before making your selection.

☒ Life sciences ☐ Behavioural & social sciences ☐ Ecological, evolutionary & environmental sciences

For a reference copy of the document with all sections, see nature.com/documents/nr-reporting-summary-flat.pdf

Life sciences study design

All studies must disclose on these points even when the disclosure is negative.

Sample size	As all experiments are simulations, they could in principle be repeated arbitrarily often. The number of repeats for each experiment is described in the Methods section. For most simulations, this sample size was arbitrarily chosen to be 25. This is sufficient because the results demonstrate qualitative differences.
Data exclusions	No exclusions
Replication	Experiments can repeated by rerunning simulations. Results do not change between simulation reruns as observed by testing simulations multiple times during development. These replications are not included in the study, but can be carried out independently through the code provided at https://github.com/jbakermans/state-space-composition.
Randomization	No experimental groups
Blinding	No group allocation, so no blinding

Reporting for specific materials, systems and methods

We require information from authors about some types of materials, experimental systems and methods used in many studies. Here, indicate whether each material, system or method listed is relevant to your study. If you are not sure if a list item applies to your research, read the appropriate section before selecting a response.

Materials & experimental systems

n/a	Involved in the study	
☒	☐	Antibodies
☒	☐	Eukaryotic cell lines
☒	☐	Palaeontology and archaeology
☒	☐	Animals and other organisms
☒	☐	Clinical data
☒	☐	Dual use research of concern
☒	☐	Plants

Methods

n/a	Involved in the study	
☒	☐	ChIP-seq
☒	☐	Flow cytometry
☒	☐	MRI-based neuroimaging

Plants

Seed stocks	*Report on the source of all seed stocks or other plant material used. If applicable, state the seed stock centre and catalogue number. If plant specimens were collected from the field, describe the collection location, date and sampling procedures.*
Novel plant genotypes	*Describe the methods by which all novel plant genotypes were produced. This includes those generated by transgenic approaches, gene editing, chemical/radiation-based mutagenesis and hybridization. For transgenic lines, describe the transformation method, the number of independent lines analyzed and the generation upon which experiments were performed. For gene-edited lines, describe the editor used, the endogenous sequence targeted for editing, the targeting guide RNA sequence (if applicable) and how the editor was applied.*
Authentication	*Describe any authentication procedures for each seed stock used or novel genotype generated. Describe any experiments used to assess the effect of a mutation and, where applicable, how potential secondary effects (e.g. second site T-DNA insertions, mosiacism, off-target gene editing) were examined.*
